# Temporal Microbial Community Dynamics in Microbial Electrolysis Cells – Influence of Acetate and Propionate Concentration

**DOI:** 10.3389/fmicb.2017.01371

**Published:** 2017-07-20

**Authors:** Ananda Rao Hari, Krishnaveni Venkidusamy, Krishna P. Katuri, Samik Bagchi, Pascal E. Saikaly

**Affiliations:** ^1^Biological and Environmental Sciences and Engineering Division, Water Desalination and Reuse Research Center, King Abdullah University of Science and Technology Thuwal, Saudi Arabia; ^2^Centre for Environmental Risk Assessment and Remediation, University of South Australia, Mawson Lakes SA, Australia; ^3^Department of Civil, Environmental, and Architectural Engineering, University of Kansas, Lawrence KS, United States

**Keywords:** microbial community dynamics, microbial electrolysis cells, acetate, propionate, *Geobacter*

## Abstract

Microbial electrolysis cells (MECs) are widely considered as a next generation wastewater treatment system. However, fundamental insight on the temporal dynamics of microbial communities associated with MEC performance under different organic types with varied loading concentrations is still unknown, nevertheless this knowledge is essential for optimizing this technology for real-scale applications. Here, the temporal dynamics of anodic microbial communities associated with MEC performance was examined at low (0.5 g COD/L) and high (4 g COD/L) concentrations of acetate or propionate, which are important intermediates of fermentation of municipal wastewaters and sludge. The results showed that acetate-fed reactors exhibited higher performance in terms of maximum current density (*I*: 4.25 ± 0.23 A/m^2^), coulombic efficiency (CE: 95 ± 8%), and substrate degradation rate (98.8 ± 1.2%) than propionate-fed reactors (*I*: 2.7 ± 0.28 A/m^2^; CE: 68 ± 9.5%; substrate degradation rate: 84 ± 13%) irrespective of the concentrations tested. Despite of the repeated sampling of the anodic biofilm over time, the high-concentration reactors demonstrated lower and stable performance in terms of current density (*I*: 1.1 ± 0.14 to 4.2 ± 0.21 A/m^2^), coulombic efficiency (CE: 44 ± 4.1 to 103 ± 7.2%) and substrate degradation rate (64.9 ± 6.3 to 99.7 ± 0.5%), while the low-concentration reactors produced higher and dynamic performance (*I*: 1.1 ± 0.12 to 4.6 ± 0.1 A/m^2^; CE: 52 ± 2.5 to 105 ± 2.7%; substrate degradation rate: 87.2 ± 0.2 to 99.9 ± 0.06%) with the different substrates tested. Correlating reactor’s performance with temporal dynamics of microbial communities showed that relatively similar anodic microbial community composition but with varying relative abundances was observed in all the reactors despite differences in the substrate and concentrations tested. Particularly, *Geobacter* was the predominant bacteria on the anode biofilm of all MECs over time suggesting its possible role in maintaining functional stability of MECs fed with low and high concentrations of acetate and propionate. Taken together, these results provide new insights on the microbial community dynamics and its correlation to performance in MECs fed with different concentrations of acetate and propionate, which are important volatile fatty acids in wastewater.

## Introduction

Microbial electrolysis cells (MECs) offer an alternative approach to effectively treat various organic waste streams with recovery of the inherent energy as hydrogen. In MECs, certain microorganisms known as exoelectrogens transport the electrons generated during the oxidation of organics in wastewater to the anode. The electrons and protons that are generated during oxidation at the anode are utilized at the cathode for H_2_ evolution reaction through the addition of minimum voltage (0.6 V) to the circuit (Logan et al., 2008). Two new and important applications of MECs are: (1) the addition of electrodes directly into anaerobic digestion (AD), in order to improve performance and increase the methane concentration in the product gas (Guo et al., 2013; Feng et al., 2015a,b; Cai et al., 2016; Liu et al., 2016). Accumulation of volatile fatty acids (VFAs) such as acetate and propionate while treating high strength wastewater, is an important concern that leads to loss in methane production and process failure of methanogenic systems such as AD (Fernandez et al., 2000; Hashsham et al., 2000; Goux et al., 2015). For example, accumulation of propionate (>20 mM) at high organic loading rates is detrimental to methanogenic systems (Pullammanappallil et al., 2001; Gallert and Winter, 2008; Ma et al., 2009). Thus, propionate removal is necessary for the stable operation of AD; and (2) integrating MECs to membrane bioreactors (MBRs), in what is referred to as anaerobic electrochemical MBR, for recovering energy and water from low strength wastewaters such as municipal wastewater (Katuri et al., 2014, 2016; Werner et al., 2016). In municipal wastewater, acetate and propionate represent the main VFAs, and their concentrations fluctuate resulting in a diverse and temporally fluctuating microbial communities. For MECs to become a viable anaerobic technology, it should adequately treat different concentrations (i.e., low and high) of acetate and propionate generated from various waste streams having different organic strength.

Anode-associated microorganisms are an important component of MECs. So far, attempts to integrate MECs to AD or MBRs have focused on the engineering aspects, reactor design and material optimization, with limited understanding of the microbial communities in the anode of MECs in response to different concentrations of acetate and propionate. Therefore, a deeper insight into the microbial community dynamics in response to different concentrations of acetate and propionate and linking it to system performance is needed. To date, most microbial studies in MECs were based on a single sampling event (typically at the end of the MEC operation) (Parameswaran et al., 2010; Lu et al., 2012b; Ruiz et al., 2014; Hari et al., 2016a,b), which provides little information on the electrochemical selection and development of microbial communities over time and how this is correlated to system performance. Nevertheless, very few studies examined the dynamics of microbial communities in MECs. For example, Lu et al. (2012a) observed a relatively similar anodic microbial community composition dominated by *Geobacter* and *Bacteroidetes* over a period of 125 days in MECs fed with acetate. Also, Kiely et al. (2011) showed that changing the operational environment from microbial fuel cell (MFC) to MEC fed with potato wastewater, dairy wastewater or acetate favors a higher relative abundance of *Geobacter* due to lack of oxygen intrusion into the system. In MFCs oxygen intrusion to the anode from the aerobic cathode affects the microbial community structure and metabolic activity of anaerobic microorganisms (Shehab et al., 2013).

To the best of our knowledge, studies understanding the temporal dynamics of microbial communities in connection to reactor performance in MECs fed with low or high concentrations of acetate or propionate have not yet been performed. Therefore, the objective of this study was to examine the temporal dynamics of microbial communities in the anodes of MECs fed with low (0.5 g COD/L) or high concentration (4 g COD/L) of acetate or propionate and relating it with reactor performance. These two different concentrations of VFAs were chosen to mimic the low and high strength wastewater containing acetate and propionate (Pullammanappallil et al., 2001; Gallert and Winter, 2008; Ma et al., 2009; Freguia et al., 2010). To address this objective, well controlled laboratory MECs were operated for a period of 70 days. Microbial communities were sampled periodically during the 70 days of batch operation and characterized by 16S rRNA gene sequencing. In addition, reactor performance in terms of current density, coulombic efficiency (CE), and substrate removal rate was continuously monitored over time.

## Materials and Methods

### Construction of MECs

Two chambered cube-shaped MECs (each chamber with a 20-mL working volume) were constructed as previously described (Hari et al., 2016a). The two chambers were separated by an anion exchange membrane (5 cm^2^; AMI 7001, Membranes International, Glen Rock, NJ, United States). A glass gas collection tube (15 mL) was attached to the top of both the anode and cathode chambers. Gasbags (0.1 L Cali -5 -Bond. Calibrate, Inc.) were connected to the top of the glass gas collection tubes to collect more volume of gas. The anodes were graphite fiber brushes (2.5 cm diameter × 2 cm long; PANEX 33 fibers, ZOLTEK Inc., St. Louis, MO, United States). The cathodes (projected surface area of 7 cm^2^) were made using carbon cloth (type B-1B, E-TEK) containing 0.5 mg/cm^2^ of Pt (Santoro et al., 2013) on the side facing the anode, and four polytetrafluoroethylene diffusion layers on another side.

### Enrichment and Operation

All MEC anodes were initially enriched in single chambered air-cathode MFCs as previously described (Call and Logan, 2008; Hari et al., 2016a) using anaerobic digester sludge (Manfouha Wastewater Treatment Plant, Riyadh, Saudi Arabia) as inoculum. Enrichment in air-cathode MFCs was done to avoid methanogenesis as oxygen intrusion through the cathode affects their growth (Hari et al., 2016a). The growth medium (pH 8.9) consisted of bicarbonate buffer (80 mM), nutrients (6.71 g/L NaH_2_CO_3_, 0.31 g/L NH_4_Cl, 0.05 g/L Na_2_HPO_4_, 0.03 NaH_2_PO_4_), Wolfe’s vitamin (10 mL/L) and trace mineral (10 mL/L) solutions (Ambler and Logan, 2011; Hari et al., 2016a,b). The medium was supplemented with two different concentrations (0.5 g COD/L or 4 g COD/L) of propionate or acetate as the energy and carbon source. The growth medium was boiled and then cooled to room temperature by sparging with N_2_:CO_2_ (80:20, vol/vol) gas mix for 30 min to remove any dissolved oxygen and was then autoclaved. The MFC anodes were transferred to individual MECs after three cycles of reproducible voltage (500 mV, over a 1 KΩ external resistor). Similar growth medium with different concentrations (0.5 g COD/L or 4 g COD/L) of propionate or acetate was used during MFC and MEC mode of operation. The duration of operation in MFC mode for the low concentration reactors (0.5 g COD/L) was ∼ 8–15 days, and ∼ 20–35 days for high concentration reactors (4 g COD/L).

A fixed voltage of 0.7 V was applied to the MECs using a power source (3645A, Array, Inc.). A total of eight MECs were operated in a parallel. Four MECs were fed only with acetate (referred to as A-reactors), and another four MECs were fed only with propionate (referred to as P-reactors). One set of duplicate MECs were operated with a low concentration of propionate (0.5 g COD/L, referred to as PL-reactors), a second set of duplicate MECs were operated with high propionate concentration (4 g COD/L, referred to as PH-reactors), a third set of duplicate MECs were operated with a low concentration of acetate (0.5 g COD/L, referred to as AL-reactors) and a fourth set of duplicate MECs were operated with high acetate concentration (4 g COD/L, referred to as AH-reactors). All reactor types (i.e., PL, PH, AL, and AH) were operated in a fed-batch mode in a temperature controlled room (30°C). When the current dropped to below 0.3 mA (PL ∼36 h/cycle; PH ∼4–5 days/cycle; AL ∼26 h/cycle; AH ∼4–5 days/cycle), the reactor solution was replaced with fresh medium and sparged with nitrogen gas (99.999%). The same growth medium was used in the anodic and cathodic compartments; however, propionate and acetate were only added to the anode medium.

### Analyzes and Calculations

The current in the circuit was determined at 20 min intervals by measuring the voltage across a resistor (10 Ω) in the circuit using a data acquisition system (Model 2700; Keithley Instruments Inc.). The current density, *I* (A/m^2^) was calculated based on the projected cathode surface area. The concentrations of propionate and acetate were analyzed by high-performance liquid chromatograph (HPLC) (Thermo Scientific, Accela, United States) equipped with a photo-diode array (210 nm) and an ultraviolet detector. An Aminex HPX-87H column (Bio-Rad Laboratories, Hercules, CA, United States) was used to separate the VFAs. Sulfuric acid (5 mM) was used as the mobile phase at a flow rate of 650 μL/min, and the pressure was maintained at 9650 kPa. The total elution time was 30 min, and each sample was measured in triplicate, and the average concentrations were reported (Lee et al., 2009). The performance of the MECs was evaluated by the current density of the reactor, *I* (A/m^2^); CE (%); substrate (propionate and acetate) removal (%) as previously described (Hari et al., 2016a,b).

### 16S rRNA Gene Sequencing

Over the course of the experiments, samples for microbial community analysis were periodically collected at different time periods (AL/PL: 0, 10, 30, 50, and 70 days; AH/PH: 0, 15/20, 35, and 70 days) from the anode and suspension of each reactor. Both biofilm and suspension samples were collected in an anaerobic glove box (Coy Laboratory Products Inc.), which was maintained under oxygen free environment. Day 0 for the anode samples represents the MFC anode that was transferred to individual MECs after three cycles of reproducible voltage (500 mV, over a 1 KΩ external resistor). The anode samples were collected by cutting about half of one round of the anode fibers using flame sterilized scissors. The graphite fiber brush anodes used in this study contained ten rounds of fiber brush (Supplementary Figure S1). At the end of the experiment, around ∼20% (four sampling events for high substrate concentrations) to 25% (five sampling event for low substrate concentrations) of the total brush surface area have been sampled for microbial analysis. The suspension samples (5 mL) were collected by pipetting into a sterile centrifuge tube followed by centrifugation at 10,000 × *g* for 8 min. The supernatant was decanted and the pellet was stored at -80°C for further analyses. Genomic DNA was extracted using the PowerSoil DNA extraction kit (MO BIO Laboratories, Inc., Carlsbad, CA, United States) following the manufacturer’s instructions. The quality (A260/A280) and quantity (A260) of the extracted genomic DNA was determined using a NanoDrop 1000 spectrophotometer (Thermo Fisher Scientific, Waltham, MA, United States).

Triplicate PCR reactions were performed for each sample in a 25 μL reaction volume using the HotStarTaq Plus Master Mix (Qiagen, Valencia, CA, United States) containing Hot Start Taq DNA polymerase (5 units/μl), 400 μM of each dNTP, 10× PCR buffer containing 3 mM MgCl_2_, 0.5 μM of each primer, and 100–200 ng of template DNA. The Bacterial (V3–V4 region) and archaeal (V3–V6 region) 16S rRNA genes were amplified using domain specific primer sets (Klindworth et al., 2012): 341F (5′-Lib-L/A-Key-Barcode-CA Linker-CCTACGGGNGGCWGCAG-3′) and 785R (5′-Lib-L/A-Key-TC Linker-GACTACHVGGGTATCTAATCC-3′) for the domain Bacteria; and 519F (5′-Lib-L/A-Key-Barcode-CA Linker- CAGCMGCCGCGGTAA-3′) and 1041R (5′-Lib-L/A-Key-TC Linker-GGCCATGCACCWCCTCTC-3′) for the domain Archaea. A unique 8-bp error-correcting barcode was used to tag each PCR product. PCR was performed using a C1000 Thermal Cycler (Bio-Rad, Hercules, CA, United States). For bacteria, the PCR conditions were as follows: initial denaturation at 95°C for 5 min, followed by 27 cycles of denaturation at 94°C for 1 min, annealing at 56°C for 1 min, extension at 72°C for 1 min and a final extension at 72°C for 7 min. For archaea, the PCR conditions were as follows: denaturation at 95°C for 5 min, followed by 35 cycles of denaturation at 94°C for 1 min, annealing at 55°C for 1 min, extension at 72°C for 1 min and a final extension at 72°C for 10 min (Klindworth et al., 2012).

The triplicate PCR products from each sample were pooled and then loaded on agarose gel and purified using the Qiaquick gel extraction Kit (Qiagen, Valencia, CA, United States) according to the manufacturer’s protocol. The concentration of the PCR products was measured with a Qubit^®^ 2.0 Fluorometer using the PicoGreen^®^ dsDNA quantitation assay (Invitrogen, Carlsbad, CA, United States). The purified barcoded amplicons from each sample were pooled in equimolar concentration and sequenced on the Roche 454 FLX Titanium genome sequencer (Roche, Indianapolis, IN, United States) according to manufacturer’s instructions.

The bacterial and archaeal 16S rRNA sequences were processed using the Quantitative Insights Into Microbial Ecology (QIIME v 1.9.0) pipeline (Caporaso et al., 2010b). Raw reads were first demultiplexed, trimmed and filtered for quality. The minimum acceptable length was set to 200 bp (Caporaso et al., 2010b). Sequences were clustered into operational taxonomic units (OTUs) at 97% sequence similarity using the uclust algorithm (Edgar, 2010). A representative sequence from each OTU was aligned using PyNAST (Caporaso et al., 2010a), and these were phylogenetically assigned to a taxonomic identity using the RDP Naive Bayesian rRNA classifier at a confidence threshold of 80% (Wang et al., 2007). Chimeric sequences were identified and removed from the aligned sequences using Chimera Slayer as implemented in QIIME. Rarified OTU tables were used to generate alpha and beta diversity metrics by normalizing to the lowest sequence read (4,100 sequences) between the samples. For alpha diversity measurements, both non-phylogenetic based metrics (observed OTUs, Shannon diversity index (H) and Chao 1 richness estimator) and phylogenetic based metric (phylogenetic diversity (PD_whole)) were calculated with QIIME. Temporal variation of bacterial community was analyzed by non-metric multidimensional scale (NMDS) using PRIMER 6 software (version 6.1.13) and PERMANOVA+ add-on (version 1.0.3). NMDS ordination was generated based on Bray–Curtis matrix (beta diversity) in QIIME. Phylogenetic diversity of abundant bacterial taxa was visualized in a heatmap using PRIMER 7 software.

### Statistical Analyses

Statistical methods were used to determine the similarity in bacterial community structure among samples. Temporal variation of bacterial community was analyzed by NMDS which was performed with Bray–Curtis matrix using QIIME and statistical software PRIMER 6 (version 6.1.13) and PERMANOVA+ add-on (version 1.0.3). Analysis of similarity (ANOSIM) was used to determine if the differences among samples is statistically significant using Bray–Curtis measure of similarity (QIIME), where the *R*-value ranges between 0 (complete similarity) to 1 (complete separation). Student’s *t*-test was performed in Microsoft Excel for all the comparisons.

### Nucleotide Sequence Accession Numbers

The 16S r RNA gene sequencing reads have been deposited in European Nucleotide Archive under the accession number PRJEB19042.

## Results

### Performance of MECs at Low and High Concentrations of Acetate and Propionate

**FIGURE 1:**
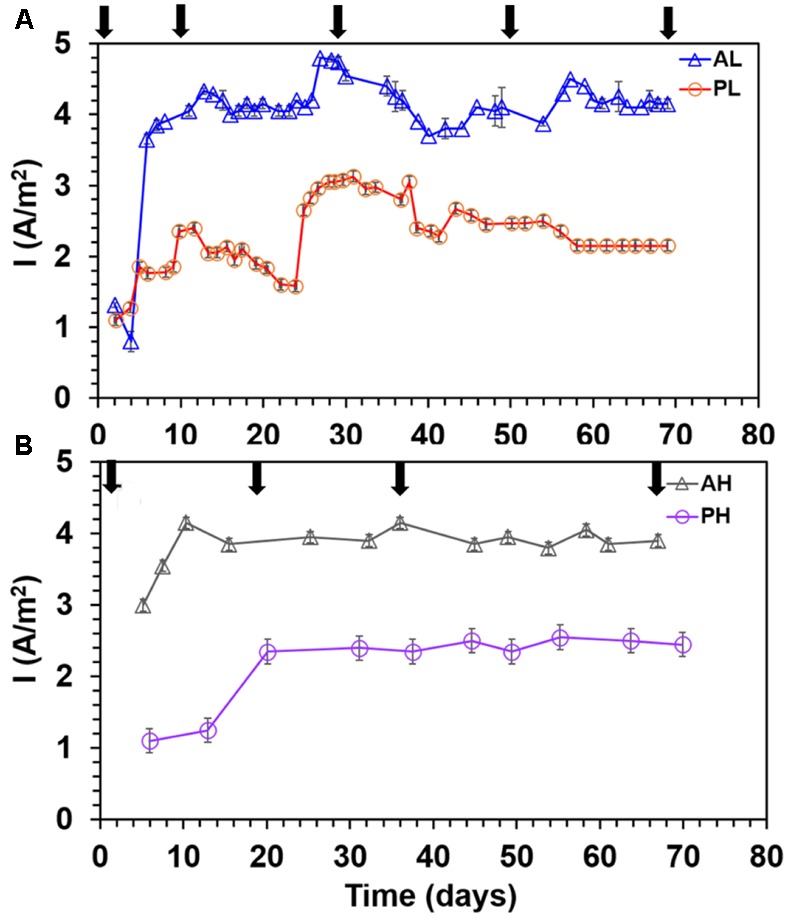
Maximum current density profile in duplicate MECs fed with low and high concentrations of acetate and propionate. **(A)** MECs fed with low substrate concentration; **(B)** MECs fed with high substrate concentration. Arrows indicate anode biofilm sampling for DNA extraction. Each data point represents the average (duplicate reactors) of the maximum current density recorded in each batch test.

A-reactors showed a higher maximum current density (4.25 ± 0.23 A/m^2^) than P-reactors (2.7 ± 0.28 A/m^2^) (*P* ≤ 0.05, Student’s *t*-test for all comparisons) irrespective of the concentrations tested (**Figure 1**). Also, A-reactors displayed a short lag time of 5–10 days (**Figures 1A,B**), whereas, P-reactors exhibited delayed startup of 10–20 days to reach maximum current density (**Figures 1A,B**). High concentration reactors showed relatively stable current density irrespective of the substrate tested (**Figure 1B**), while it was dynamic in the low concentration reactors (**Figure 1A**). For instance, in PL reactors, the maximum current density increased from 1 A/m^2^ on day 2 to 2.3 A/m^2^ on day 10 followed by a decrease in the maximum current density of 1.5 A/m^2^ on day 22. Then maximum current density of 3.3 A/m^2^ was reached on day 23 and remained steady until day 35 of operation (**Figure 1A**). Furthermore, it relatively decreased to 2.2 A/m^2^ in the next 4 days and eventually reached a stable electrical current of 2.5 A/m^2^ until the termination of the experiment on day 70. Cutting a portion of the anode fibers at each sampling event caused a decline in electrical current production in the low concentration reactors irrespective of the substrate tested (**Figure 1A** and Supplementary Figures S2A,C). However, high concentration reactors showed stable performance despite of the sampling event (**Figure 1B** and Supplementary Figures S2B,D). It should be noted that the MECs were operated at a fixed voltage of 0.7 V and the resulting anode potential was -0.23 ± 0.09 V vs. SHE (P-reactors) and ∼-0.15 ± 0.1 V vs. SHE (A-reactors).

**FIGURE 2:**
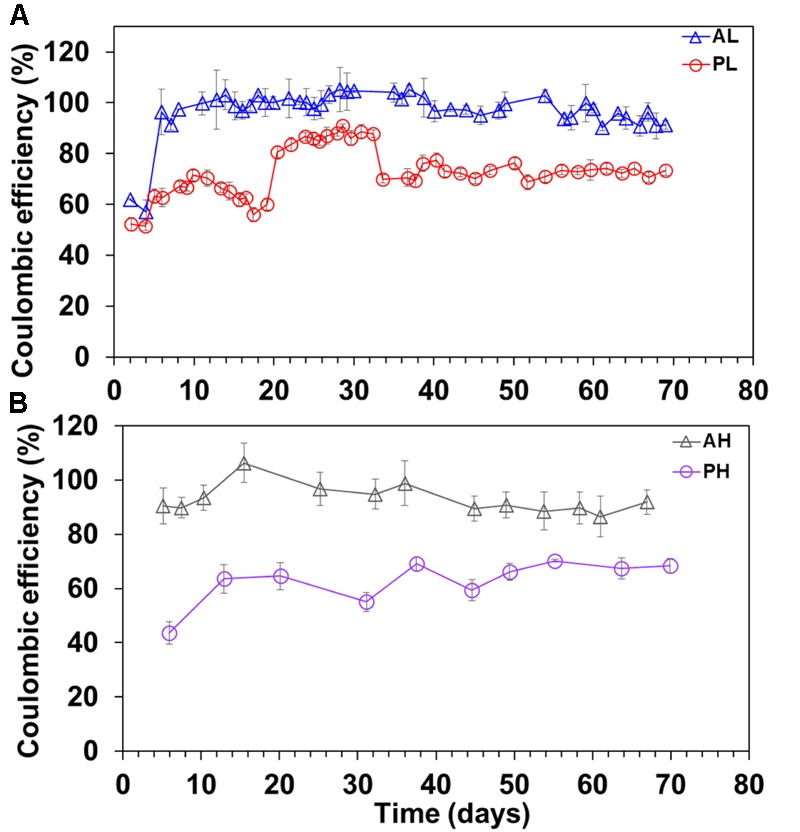
Coulombic efficiency (CE) in duplicate MECs fed with low and high concentrations of acetate or propionate. **(A)** MECs fed with low acetate concentration (AL); **(B)** MECs fed with high acetate concentration (AH); **(A)** MECs fed with low propionate concentration (PL); **(B)** MECs fed with high propionate concentration (PH). The values correspond to the average of the duplicate reactors. Each data point represents the average (duplicate reactors) of the CE recorded in each batch test.

The average CE (%) for the whole period (i.e., 70 days) of operation of the MECs was: AL (96.7 ± 9%), AH (93 ± 5%), PL (73 ± 9%), and PH (63 ± 8%) (**Figure 2**). The A-reactors yielded higher CE (95 ± 8%) than the P-reactors (68 ± 9.5%) (*P* ≤ 0.05). Particularly, the CE of few batches of A-reactors were >100% (**Figure 2**). Furthermore, the CE in the A-reactors was more stable in comparison to the P-reactors (**Figure 2**). Irrespective of the substrate tested, low concentration reactors (AL/PL: 86 ± 10%) produced a relatively higher CE than the high concentration reactors (AH/PH: 78 ± 7.5%) (*P* ≤ 0.05) (**Figure 2**).

**FIGURE 3:**
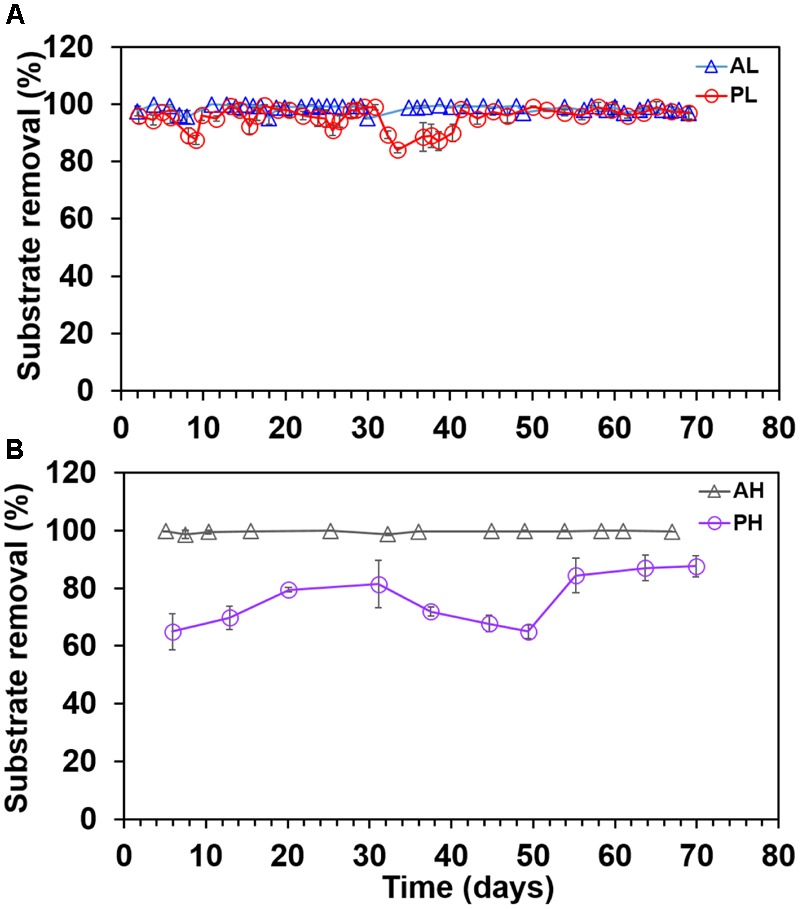
Substrate removal trend in duplicate MECs fed with low and high concentrations of acetate and propionate. **(A)** MECs fed with low acetate concentration (AL); **(B)** MECs fed with high acetate concentration (AH); **(A)** MECs fed with low propionate concentration (PL); **(B)** MECs fed with high propionate high (PH). Each data point represents the average (duplicate reactors) of substrate removal recorded in each batch test.

Substrate removal was nearly complete in A-reactors (98.8 ± 1.2%) with no significant difference between AL and AH-reactors (*P* > 0.2) (**Figures 3A,B**), whereas, variable percentage of substrate removal was noticed in P-reactors (PL: 93 ± 8.6; PH: 75 ± 14) (**Figures 3A,B**). The substrate removal rates (g COD)/L/Day were, for AL (0.33 ± 0.05), AH (0.90 ± 0.14), PL (0.27 ± 0.06), and PH (0.46 ± 0.05) reactors. Also, the pH of the medium was 7.6 ± 0.5 in A- and P-reactors at the end of fed-batch cycle.

The error bars in **Figure 2** were relatively bigger than **Figures 1, 3** because several data points (every 20 min of batch time) from each batch test were used to calculate the CE, whereas one data point was used to determine the maximum current density and substrate removal for each batch cycle.

### Microbial Community Analysis

16S rRNA gene sequencing was used to characterize the bacterial and archaeal communities of anode and suspension samples from duplicate MEC reactors (AL, AH, PL, and PH). A total of 1,066,983 (bacteria) and 503,327 (archaea) high quality reads (average length of ∼400 bp) were obtained after denoising, quality filtering, and removal of chimeric sequences. For downstream analysis, OTUs with 97% sequence identity threshold were used.

#### Bacterial Community Diversity

For alpha diversity measures, we subsampled the dataset to an even depth of 4,100 sequences across the samples to remove inherent heterogeneity of sampling depth. This number was chosen, as it corresponds to the lowest number of sequence reads detected. The diversity values across the anode and suspension samples of A- and P-reactors ranged as follows: observed OTUs (74-1225), Chao 1 (216-3465), Shannon diversity index (H; 2.2-6.9), and phylogenetic diversity (PD; 12-79) (**Tables 1, 2**). The bacterial diversity was higher in the anode and suspension of P-reactors than A-reactors based on observed OTUs, Chao 1, PD and H (**Tables 1, 2**). Also, the high concentration-fed reactors (AH/PH) revealed a higher diversity than low concentration-fed reactors (AL/PL) (**Tables 1, 2**). Time series analysis of A-reactors indicated that bacterial diversity of anode and suspension samples was higher on day 0 (i.e., MFC mode of operation) and considerably reduced at the end of the experiment (i.e., day 70) (**Table 1**). Likewise, the P-reactors, particularly, PH-reactors showed a similar trend on day 0 (anode and suspension), and relatively decreased at the end of the experiment (**Table 2**). In contrast, PL-reactors (anode and suspension) revealed that the bacterial diversity was higher on day 0 and significantly reduced with time until day 50 (*P* < 0.05), followed by an increase in diversity on day 70 (**Table 1**). No clear trend could be observed for propionate-fed MECs when comparing alpha diversity between anode and suspension samples. For example, in PL reactors, the diversity of suspension samples was higher than anode samples, whereas in PH MECs diversity was higher in the anode than suspension samples for all the sampling periods (**Table 2**). In contrast, a clear trend in diversity was observed in AL and AH reactors, where diversity was higher in suspension than anode samples for all the sampling periods except day 70 (**Table 1**).

**Table 1:** Measures of alpha diversity of bacterial phylotypes in acetate reactors.

Sample	Chao1	Observed OTUs	PD index	Shannon diversity index
A_AL_0	808 ± 100	238 ± 12	29 ± 1.7	5.3 ± 0.12
A_AL_10	216 ± 71	74 ± 4	12 ± 0.8	2.18 ± 0.08
A_AL_30	262 ± 73	79 ± 6	12 ± 0.8	2.39 ± 0.08
A_AL_50	223 ± 74	85 ± 8	13 ± 0.8	2.82 ± 0.07
A_AL_70	274 ± 41	119 ± 4	17 ± 0.8	5.05 ± 0.05
S_AL_0	547 ± 72	213 ± 9	26 ± 1.2	5.64 ± 0.09
S_AL_10	274 ± 56	95 ± 4	12 ± 0.8	3.69 ± 0.06
S_AL_30	271 ± 80	102 ± 8	13 ± 1	3.88 ± 0.07
S_AL_50	298 ± 88	107 ± 6	16 ± 1	4.39 ± 0.09
S_AL_70	128 ± 9	85 ± 1	12 ± 0.1	4.33 ± 0.01
A_AH_0	906 ± 169	244 ± 15	30 ± 1.6	5.54 ± 0.11
A_AH_15	745 ± 125	217 ± 10	24 ± 1.6	4.99 ± 0.10
A_AH_35	404 ± 83	159 ± 7	21 ± 0.9	4.9 ± 0.09
A_AH_70	559 ± 81	188 ± 8	21 ± 1.1	4.78 ± 0.07
S_AH_0	628 ± 107	225 ± 9	27 ± 1.4	5.63 ± 0.06
S_AH_15	765 ± 126	235 ± 8	26 ± 1.2	5.54 ± 0.07
S_AH_35	551 ± 109	191 ± 8	22 ± 0.7	5.74 ± 0.05
S_AH_70	435 ± 28	181 ± 5	18 ± 0.5	4.51 ± 0.06

*A, anode; S, suspension.*

**Table 2:** Measures of alpha diversity of bacterial phylotypes in propionate reactors.

Sample	Chao1	Observed OTUs	PD index	Shannon diversity index
A_PL_0	1948 ± 125	560 ± 20	46 ± 2	6.03 ± 0.07
A_PL_10	1364 ± 225	385 ± 12	33 ± 2	4.92 ± 0.11
A_PL_30	1062 ± 122	341 ± 11	30 ± 1	4.18 ± 0.07
A_PL_50	1370 ± 82	399 ± 4	34 ± 0.5	5.03 ± 0.02
A_PL_70	1820 ± 121	543 ± 7	44 ± 0.7	6.4 ± 0.02
S_PL_0	2511 ± 347	641 ± 10	51 ± 2	6.8 ± 0.04
S_PL_10	1467 ± 100	446 ± 6	38 ± 0.7	5.9 ± 0.09
S_PL_30	1046 ± 131	365 ± 10	31 ± 1	4.74 ± 0.06
S_PL_50	1664 ± 24	568 ± 1	44 ± 0.05	6.79 ± 0.004
S_PL_70	1969 ± 252	524 ± 15	45 ± 2	6.12 ± 0.14
A_PH_0	3465 ± 265	1225 ± 14	75 ± 2	6.5 ± 0.04
A_PH_20	2858 ± 172	1051 ± 18	65 ± 1	6.2 ± 0.06
A_PH_35	2415 ± 190	887 ± 15	57 ± 0.8	5.5 ± 0.04
A_PH_70	2395 ± 227	899 ± 18	58 ± 1.5	5.6 ± 0.04
S_PH_0	2496 ± 117	1065 ± 15	65 ± 1	6.84 ± 0.07
S_PH_20	2885 ± 14	1032 ± 1	62 ± 0.03	6.26 ± 0.04
S_PH_35	2029 ± 110	899 ± 23	54 ± 2	6.81 ± 0.04
S_PH_70	1607 ± 171	625 ± 15	41 ± 1	4.42 ± 0.02

*A, anode; S, suspension.*

#### Bacterial Community Structure

**FIGURE 4:**
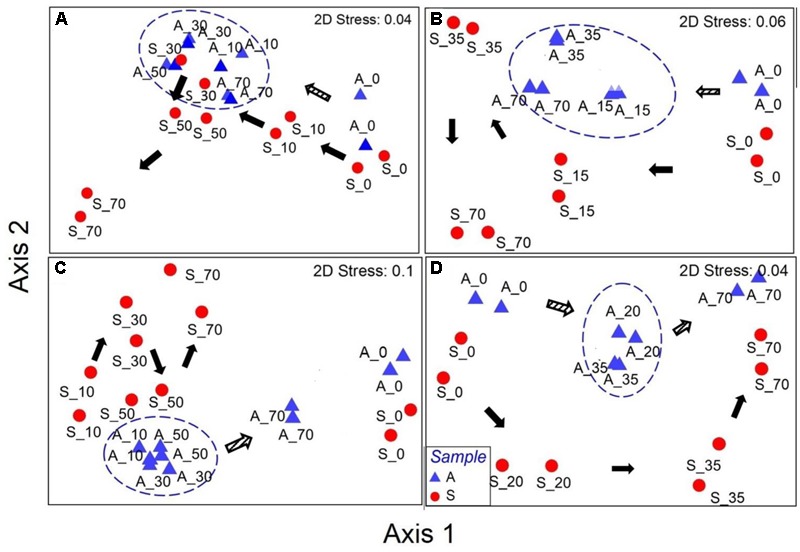
Non-metric multidimensional scaling plots based on Bray-Curtis distance for **(A)** AL, **(B)** AH, **(C)** PL, and **(D)** PH reactors. A, anode and S, suspension. The numbers after the symbols represent the sampling day (AL/PL: days 0, 10, 30, 50, and 70; AH/PH: days 0, 15/20, 35, and 70). Duplicate samples are shown as individual data points in the plots. Circles were manually drawn to represent the clusters of converging anodic bacterial community over time. Arrows indicate the pattern of evolution in the microbial community from day 0. Unfilled and filled arrows represent the anode and suspension samples, respectively.

**FIGURE 5:**
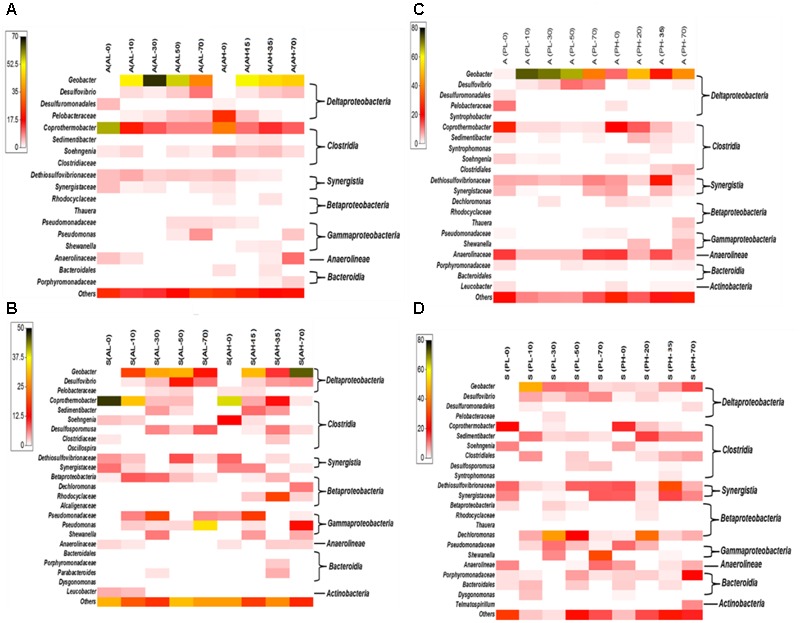
Heat map distribution of bacterial phylotypes classified to the lowest level possible (class, order, family or genus) for A- and P- reactors: anode of A-reactors **(A)**, suspension of A-reactors **(B)**, anode of P-reactors **(C)** and suspension of P-reactors **(D)**. A, anode and S, suspension. Bacterial phyla representing less than 1% of the relative abundance are classified as others. The taxa level shown on the left-hand side of the panel represents the lowest classification level possible (order, family or genus), while the taxa level on the right-hand side represents class. Scale on top left-side of each panel is relative abundance (%). Each cell represents the average of duplicate reactors. Each column represents a specific sampling period (AL/PL: days 0, 10, 30, 50, and 70; AH/PH: days 0, 15/20, 35, and 70). Day 0 represents the MFC anode that was transferred to individual MECs after three cycles of reproducible voltage.

Non-metric multidimensional scaling analysis based on Bray–Curtis revealed that all the samples (anode and suspension) had a gradual succession away from the initial conditions and a relatively similar pattern of succession was observed between low and high concentration-fed reactors (**Figure 4**). However, the development and succession paths of anodic bacterial communities were different between A- and P-reactors (**Figure 4**). For example, A-reactors showed that all the anodes (AL: 10, 30, 50, and 70 days; AH: 15, 35, and 70 days) were clustered together and distantly away from the initial anode samples (0 days) (**Figures 5A,B**). In contrast, P-reactors showed that the anode samples (PL: 10, 30, and 50; PH: 20 and 35 days) were clustered together and away from the anode sample at day 70 (**Figures 4C,D**). Also, NMDS results showed that the temporal variation in the bacterial community structure was higher for the suspension samples (A- and P- reactors) than the anode samples as can be seen by their wider distribution in the ordination plot (**Figure 4**). Temporal variation in the microbial community structure within the suspension samples (different sampling points) was significant (*p* = 0.0001, *R* = 0.6357) as confirmed by ANOSIM. Whereas, lower temporal variation was obtained in the microbial community structure within biofilm samples (*p* = 0.003, *R* = 0.2243). Statistically significant difference between the anode and suspension microbial community structure was found using ANOSIM (*p* = 0.0001, *R* = 0.7283).

#### Bacterial Community Composition and Dynamics

A heatmap was generated to represent the various phylotypes identified from the A- and P-reactors down to the lowest classification level possible (class, order, family, or genus) (**Figure 5** and Supplementary Tables S1–S4). Highly abundant phylotypes belonging to the different detected bacterial classes are discussed below:

##### Deltaproteobacteria

Four phylotypes belonging to the class *Deltaproteobacteria* were relatively abundant in the A- and P-reactors (**Figure 5** and Supplementary Tables S1–S4). Among *Deltaproteobacteria, Geobacter* was highly dominant over time (10 to 70 days) in the anode of A- and P-reactors (**Figure 5A,C**). *Geobacter* was detected in very low fraction (<1%) in the A-reactors at day 0. However, it become highly dominant (AL: 52 ± 13%; AH: 49 ± 4%) over time (10 to 70 days) (**Figure 5A**). The relative abundance of *Geobacter* in the anode of P-reactors was different between low and high concentration-fed reactors (**Figure 5C**). For example, its relative abundance was 1.4 ± 0.5% at day 0 of PL-reactors, and it significantly increased to reach 70 ± 3.4% between days 10 and 50 days followed by a significant decrease to 39 ± 2.3% (*P* < 0.05) on day 70 (**Figure 5C**). In contrast, PH-reactors revealed that the relative abundance of *Geobacter* was dynamic over time (**Figure 5C**). For instance, the relative abundance of *Geobacter* was 11.5 ± 1.8% on day 0 and increased to 48.7 ± 6.1% on day 20 (**Figure 5C**). Following operation at day 35, the relative abundance of *Geobacter* was drastically reduced to 23.3 ± 2.7%, but it increased again to reach 41.9 ± 6.2% on day 70. The higher abundance of *Geobacter* at day 0 in the PH reactors was due to the relatively longer period of operation (35 days) compared to AL (8 days), AH (20 days), and PL (15 days) reactors. Collectively, the above results show that electrochemical selection of *Geobacter* significantly enhanced in MEC mode of operation as evidenced by the significant increase in their relative abundance after day 0 (Supplementary Tables S1–S4). The suspensions of A- and P-reactors revealed that the relative abundance of *Geobacter* was dynamic over time (3–45%) (**Figures 5B,D**). *Desulfovibrio* was relatively identified in all the samples of A- and P-reactors (1–14%) (**Figure 5**). *Pelobacteraceae* was relatively more abundant in the anode of A-reactors (1–23%) than P-reactors (2–11%). *Desulfuromonadales* was present during the early stages of A- and P-reactors (1–5%) (**Figure 5**).

##### Clostridia

Seven phylotypes belonging to the class *Clostridia* were frequently observed in all the samples of A- and P-reactors (**Figure 5**). *Copothermobacter* was highly abundant in A-reactors than P-reactors (**Figure 5**). Specifically, it was abundant during earlier stages of reactor operation (between day 0 and 20) (A-reactors: 45 ± 11%; P-reactors: 22 ± 3.5%) and was significantly reduced during later stages of reactor operation (days 50 and 70) (A-reactors: 7.7 ± 5%; P-reactors: 2.5 ± 1.7%) (*P* < 0.05) (**Figures 5A**,). *Sedimentibacter* was relatively abundant over time in the P-reactors (1–14%) than A-reactors (2–7%). Particularly, it was more prevalent in the suspension than the anode of A- and P-reactors. *Sohengenia* was present in the anode of A-reactors over time (1–6%), and it was present only during the early stages of operation in the P-reactors (1–9%). *Syntrophomonas* was present only in the anode of PH-reactors (2%). *Desulfosporomusa* was observed only in the suspension. Specifically, it was more prevalent in the A- reactors (1–8%) than P-reactors (1–4%) (**Figure 5**).

##### Synergistia

Two different phylotypes (*Dethiosulfovibrionaceae* and *Synergistaceae*) belonging to the class *Synergistia* were consistently observed in the A- and P-reactors over time (**Figure 5**). Specifically, *Dethiosulfovibrionaceae* was dominant throughout the operation of the P-reactors (anode and suspension) than A-reactors (**Figures 5C,D**). In addition, *Synergistaceae* was found to be dominant in the suspension than the anode of A- and P-reactors (**Figures 5B,D**).

##### Betaproteobacteria

Four different phylotypes belonging to the class *Betaproteobacteria* were observed in the samples of A- and P-reactors (**Figure 5**). *Dechloromonas* was considerably abundant in the suspension of P-reactors over time (2–41%). *Rhodocyclaceae* was relatively abundant (3–19%) in the suspension of AH-reactors over time (**Figure 5B**).

##### Gammaproteobacteria

Three different phylotypes (*Pseudomonas, Pseudomonadaceae*, and *Shewanella*) belonging to the class *Gammaproteobacteria* were observed in the A- and P-reactors (**Figure 5**). Specifically, *Pseudomonas* and *Pseudomonadaceae* were relatively dominant over time in the A-reactors than the P-reactors (**Figure 5**). *Shewanella* was noticed as a minor fraction in the A- and P-reactors (**Figure 5**). However, it was more prevalent (29 ± 2.5%) in the suspension of PL-reactors on day 70 (**Figure 5D**).

##### Bacteroidia

Four different phylotypes of the class *Bacteroidia* (*Dysgonomonas, Bacteroidales, Porphyromonadaceae*, and *Parabacteroides*) were noticed in the A- and P-reactors (**Figure 5**). *Porphyromonadaceae* and *Bacteroidales* were relatively more dominant in the P-reactors (7 ± 4%) than the A-reactors (3 ± 0.4%) (**Figure 5**), particularly it was more prevalent in the suspension than the anode of the P-reactors (**Figure 5D**). *Parabacteroides* was present only in the suspension of A-reactors (**Figure 5B**).

##### Anaerolineae

Only one phylotype of the class *Anaerolineae* namely *Anaerolinaceae* was observed in the A- and P-reactors (**Figure 5**). It was more abundant (3–16%) in the P-reactors than the A-reactors. Specifically, it was more dominant in the anode than the suspension of P-reactors (**Figures 5C,D**).

#### Archaeal Community Composition and Dynamics

Archaeal 16S rRNA gene sequences revealed the dominance of *Methanobacteriaceae* (74.5 ± 13%) (hydrogenotrophic methanogens) in all the samples (anode and suspension) of A- and P-reactors (Supplementary Figure S3). Particularly, the most abundant genera was *Methanobacterium* in all the samples of A-reactors (65 ± 13%) (Supplementary Figures S3A,B) and the anode of P-reactors (57 ± 15%) (Supplementary Figure S3C). Whereas, *Methanobrevibacter* was more abundant in the suspension of P-reactors (61 ± 12%) (Supplementary Figure S3D).

Temporal analysis of archaeal 16S rRNA gene sequences of A-reactors revealed the predominance of *Methanobacterium* (65 ± 13%) over time followed by *Methanobrevibacter* (10 ± 6%), *Thermoplasmata* (*WCHD3-02*) and *Crenarchaeota* (*MCG*) (excluding days 0 and 10 of AL-reactor samples which failed to amplify) (Supplementary Figures S3A,B). Also, A-reactors contained a minor fraction of unclassified *Methanobacteriaceae, Methanospirillum, Methanosarcina*, and *Methanosaeta.* In contrast, temporal analysis of the archaeal community of P-reactors displayed that the anodes were dominated by the hydrogenotrophic methanogens, *Methanobacterium* (PL: 51 ± 12%: PH: 64 ± 18%) followed by acetoclastic methanogens, *Methanosaeta* (PL: 10 ± 6%: PH: 14 ± 4%) (Supplementary Figure S3C). In addition, other sub-dominant communities were observed namely unclassified *Methanobacteriaceae, Methanobrevibacter, Methanospirillum, Methanosarcina*, and *Thermoplasmata* (WCHD3-02). *Methanobrevibacter* (PL: 64 ± 15%; PH: 60 ± 14%) was dominant in the suspension samples of P-reactors followed by *Methanobacterium* (PL: 12 ± 8%: PH: 25 ± 15%) (Supplementary Figure S3D).

## Discussion

The results gathered in this study demonstrated that A-reactors produced greater performance than the P-reactors in terms of current density, CE, and substrate removal efficiency regardless of the concentrations tested (**Figures 1–3**). Acetate in the A-reactors can be directly consumed by *Geobacter* for electricity generation, whereas, in the P-reactors, electricity generation requires microbial partnership between propionate degraders and intermediate consumers (e.g., *Geobacter*) resulting in more loss of electrons to various other competing electron sinks [biomass synthesis and production of soluble microbial products (SMPs)] as previously described (Lee et al., 2008; Ishii et al., 2014; Vanwonterghem et al., 2014; Hari et al., 2016a,b). In addition, A-reactors produced CEs greater than 100% in some of the batches (**Figures 2A,B**) possibly due to (1) H_2_ cycling from the cathode to the anode (Lee et al., 2009; Siegert et al., 2014; Zhu et al., 2014); (2) oxidation of intracellular biopolymers such as polyhydroxyalkanoates (Koch et al., 2014); or (3) utilization of stored energy in the cells (Siegert et al., 2014). In general, high concentration reactors (AH/PH) exhibited lower reactor performance in terms of maximum current density and CE (**Figures 1, 2**) than the low concentration reactors (AL/PL), possibly due to loss of electrons to other competing electron sinks as described previously (Hari et al., 2016a,b). Nevertheless, high concentration reactors (AH/PH) showed stable (reproducible) performance, despite repeated disturbance of the anode biofilm over time for sampling. The effect of disturbance caused by frequent sampling was more pronounced in AL and PL reactors where reduction in current density was observed followed by recovery to maximum current density in a short period (**Figure 1** and Supplementary Figure S2). This suggests the self-optimization of MECs for attaining stable (reproducible) performance after disturbance.

Regardless of the substrate and concentrations tested, the anodic microbial community structure between duplicate MEC reactors was similar at each sampling event (**Figure 4**). During MEC mode of operations, a relatively similar anodic bacterial community structure was observed in the A-reactors over time regardless of the concentration tested, whereas in the P-reactors the bacterial communities at day 70 were clustered separately from the remainder of the samples (**Figure 4**). The succession observed in the anode of A- and P-reactors where dominance of *Copothermobacter* and *Anaerolinaceae* on day 0 (MFC mode of operation) was replaced by *Geobacter* on day 10, and the eventual dominance of *Geobacter* over time (days 10 to 70) (**Figure 5**) suggests that operation in MEC mode not only influenced the dominance of *Geobacter* but also decreased the bacterial diversity over time (10–70 days) (**Tables 1, 2**) (Kiely et al., 2011; Lu et al., 2012a). It should be noted that the dominance of *Geobacter* was also observed on the anode of MECs fed with domestic wastewater (Heidrich et al., 2014). At the end of MEC operation (day 70), AL and PL reactors showed a decrease in the relative abundance of *Geobacter*, but it remained the predominant community, accompanied by an increase in the relative abundance of several phylotypes (e.g., *Desulfovibrio* and *Pseudomonas* in AL reactors and *Dethiosulfovibrionaceae, Synergistaceae*, and *Anaerolinaceae* in PL reactors) (**Figures 5A,C**), however, the current density remained stable (**Figure 1**). An earlier study on pilot scale MEC treating domestic wastewater showed in addition to the *Geobacter*, the dominance of the hydrolytic microorganism *Synergistia (Dethiosulfovibrionaceae*), which likely resulted in more positive impact on the reactor performance (Heidrich et al., 2014). *Anaerolineae* was found as a predominant group in the electrode of integrated MEC-Anaerobic digestion system (Liu et al., 2016) and was also detected as a dominant organism in the anode of MFCs fed with the root exudates of rice field soil (Cabezas et al., 2015), suggesting that it likely played a role as an exoelectrogen and/or fermenter; however, its role in this study is unclear. In the case of the PH-reactors, a stable trend in the maximum current density with each cycle was observed despite varying microbial community structure over a time (days 20–70) (**Figure 5C**). These results suggest that functional stability was maintained despite changes in community structure. Previous studies reported that the anodic microbial communities in MFCs are flexible and can self-select and self-optimize to maintain functional stability (Ishii et al., 2012; Koch et al., 2014). In the current study, the predominance of *Geobacter* on the anode over time (**Figures 5A,C**) was essential for maintaining relatively stable current density pattern (**Figure 1**) of MECs fed with low or high concentrations of acetate or propionate. This suggests that the presence of members of the genus *Geobacter* on the anode is likely essential for successful implementation of MECs for full-scale anaerobic treatment of low and high strength wastewater.

Although, the current study was not designed to test spatial variation in microbial community structure, we cannot rule out that some of the variations observed in the microbial community structure over time could be due to spatial variation (i.e., different sampling positions). Nevertheless, previous studies reported no spatial variation in the microbial community structure on both planar and volumetric electrodes. For example, Vargas et al. (2013) showed that the anodic microbial community at different locations on planar (carbon cloth) and volumetric anodes (fiber brush anodes) was homogenous based on 16S rRNA gene sequencing. The anode fiber brush used in the current study was similar to the one used by Vargas et al. (2013). Dennis et al. (2013) specifically designed a reactor setup to address if changes in microbial diversity observed over time in a bioelectrochemical system can be related to community development rather than spatial variation within the reactor. Their 16S rRNA genes sequencing results revealed no spatial variation in the diversity of microbial communities associated with different planar electrodes within a single time point. Using fluorescence *in situ* hybridization (FISH), Kiely et al. (2011) showed that *Geobacter sulfurreducens* was homogenously dispersed on the anode fibers of a potato wastewater fed MECs.

In addition, the microbial community composition and structure was compared between the anode biofilm and suspension samples. The bacterial community structure in the suspension of A- and P-reactors was highly dynamic compared to the biofilm samples as revealed by NMDS (**Figure 4**) and ANOSIM. However, similar bacterial composition was observed between the anode and suspension samples, where the dominant members on the anode were also present in suspension (Supplementary Tables S1–S4). This similarity in bacterial composition between the biofilm and suspension samples could be due to biofilm detachment. This was supported by the presence of high abundance of *Geobacter* (3–45%) in the suspension samples (**Figures 5B,D** and Supplementary Tables S2, S4). It is well known that members of the genus *Geobacter* are strongly associated with the anode, and their presence in solution was due to biofilm detachment. It should be noted, that at the end of each batch cycle the anolyte solution was emptied and replaced with fresh autoclaved solution, and current generation was immediately observed for both acetate- and propionate-fed MECs. Taken together, these results suggest that the main microbial functions (exoelectrogenesis and fermentation) were taking place at the anode and the slight turbidity observed in the anolyte solution of the acetate- and propionate-fed reactors was possibly due to biofilm detachment.

**FIGURE 6:**
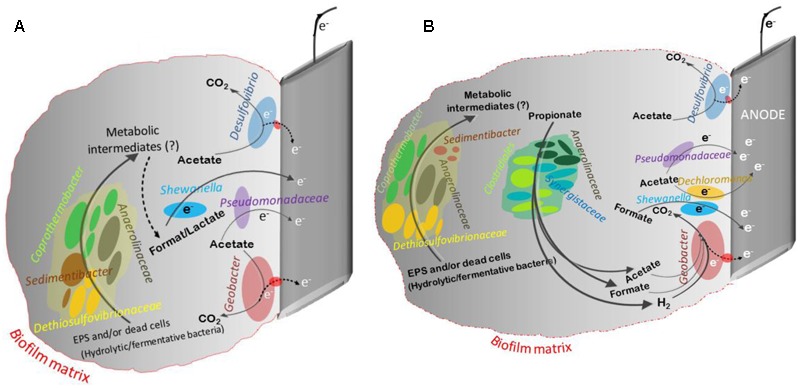
A schematic describing the potential key members in the community and their interaction in the anode of MECs fed with acetate **(A)** or propionate **(B)**. This schematic was developed based on the available information from the literature on the microbial communities. Members of *Geobacter, Shewanella* (Luo et al., 2017), *Desulfovibrio and Pseudomonadaceae* spp. are known to use the anode as their electron acceptor (Koch and Harnisch, 2016). The presence of complex carbohydrates and proteins in the extracellular polymeric substances (EPS) and/or endogenous decay of dead cells in the anodic biofilm matrix can act as a source of substrate for various hydrolytic/fermentative bacterial communities, like *Anaerolinaceae, Sedimentibacter, Dethiosulfovibrionaceae*, and *Coprothermobacter*. This process might lead to the production of unknown metabolic intermediates (e.g., acetate, propionate, formate, lactate, H_2_, etc.), and these intermediates can act as an electron and carbon source for the growth of other microbial communities (fermenters and exoelectrogens). For example, members of C*oprothermobacter* spp. were found to have strong intracellular and extracellular protease activity capable of protein and peptide degradation. Additionally, they were identified in anaerobic systems as important hydrogen producers (Tandishabo et al., 2012). Development of mixed populations of species belonging to *Firmicutes, Synergistetes, Coprothermobacteria*, and *Anaerolineae* were observed in microbial fuel cell (MFC) fed with acetate (Fu et al., 2013) and of root exudate-driven MFCs (Cabezas et al., 2015). Members of *Clostridia, Synergistia*, and *Anaerolineae* were reported to be propionate oxidizers and were abundant in propionate and acetate fed MECs, propionate enriched soils, and anaerobic digester sludge (Chauhan et al., 2004; Kragelund et al., 2007; Yamada et al., 2007; Ito et al., 2011; Lesnik and Liu, 2014; Ruiz et al., 2014; Cabezas et al., 2015).

Analysis of 16S rRNA gene pyrosequencing data revealed that the classical propionate degraders such as *Syntrophobacter* spp., *Smithella* spp., and *Pelotomaculum* spp. that are typically present in methanogenic systems were not detected on the anode and suspension in the current study. It is possible that the conditions in the anode chamber favored the presence of other propionate degraders. In the current study, P-reactors were dominated by diverse phylotypes belonging to *Clostridia, Synergistia*, and *Anaerolineae* (**Figures 5B,D**). Members of these classes were reported to be propionate oxidizers and were abundant in propionate and acetate fed MECs, propionate enriched soils, and anaerobic digester sludge (Chauhan et al., 2004; Kragelund et al., 2007; Yamada et al., 2007; Ito et al., 2011; Lesnik and Liu, 2014; Ruiz et al., 2014; Cabezas et al., 2015). A schematic diagram based on 16S rRNA gene sequencing data was generated describing the potential key members in the community and their interaction in the anode of MECs fed with acetate or propionate (**Figure 6**). It should be noted that in-depth community analysis using 16S rRNA gene pyrosequencing allows for speculation about possible interactions between different members in the community when utilizing an organic substrate (Aracic et al., 2014). However, it does not provide information on the metabolically active members in the community. Alternatively, stable isotope probing (SIP) with labeled propionate combined with 16S rRNA gene sequencing should be used in future studies to provide a better insight into the carbon flow during propionate oxidation and to allow the identification of metabolically active members in the community (i.e., linking phylogeny to function) that are involved in propionate oxidation (Dumont and Murrell, 2005). Also, additional insights into the physiology of electrode communities can be obtained using metatranscriptomics (Ishii et al., 2013; Aracic et al., 2014).

In terms of substrate removal, AL, AH, and PL showed nearly complete removal of substrate (97 ± 2%), whereas substrate removal was 75 ± 14% in the PH-reactors (**Figure 3B**). In methanogenic systems, propionate oxidation is highly endergonic (+72 kJ/mole) and performed by syntrophic consortia of microorganisms (Stams and Plugge, 2009). In such environments, acetogenic bacteria oxidize propionate to acetate and hydrogen (or formate), which are then utilized by acetoclastic (acetate) and hydrogenotrophic methanogens (H_2_ or formate) to produce CH_4_ or CO_2_ (Boone and Xun, 1987). In general, accumulation of high concentrations of propionate (>20 mM) is detrimental to AD processes (Pullammanappallil et al., 2001; Gallert and Winter, 2008; Ma et al., 2009). In MECs, complete removal of propionate at elevated substrate concentration (36 mM) requires microbial partnership between fermenters, hydrogenotrophic methanogens and *Geobacter* (Hari et al., 2016b). Both *Geobacter* and hydrogenotrophic methanogens consumed the intermediates (acetate, H_2_, and formate) generated by fermenters, and kept their concentrations low resulting in more energetically favorable fermentation, and hence complete removal of propionate (Hari et al., 2016b). In the current study, the incomplete degradation of propionate in PH reactors was possibly due to lack of hydrogenotrophic methanogenesis, which was not a major sink (data not shown) of electrons. The lack of methanogenesis in the current study was possibly due to the fact that the MEC anodes were initially enriched in single chamber air-cathode MFCs, where oxygen intrusion through the cathode might have affected the growth of methanogens. In another study, we operated the P-reactors under MEC (oxygen free environment) mode from the start of the experiment, and this provided a suitable environment for the enrichment of hydrogenotrophic methanogens resulting in methane being an important sink (Hari et al., 2016b).

While the use of an applied voltage in the current study was useful for understanding the temporal dynamics of microbial communities in connection to reactor performance in MECs fed with low or high concentrations of acetate or propionate, the anode potentials in the reactors were not controlled. However, at all tested conditions the variation in the anode potential was low (∼-0.15 ± 0.1 V to -0.23 ± 0.09 V vs. SHE) resulting in relatively similar anodic microbial community composition but with varying relative abundance. It is known that operating MECs as set anode potential (SAP) can influence the anodic microbial community structure (Torres et al., 2009; Hari et al., 2016b). For example, in propionate-fed MECs higher microbial diversity was observed at more positive SAP (0.25 V vs. SHE) than lower SAPs (0 V and -0.25 V). Also, similar dominant genera (*Geobacter, Smithella*, and *Syntrophobacter*) were observed on the anode of all tested SAPs, but their relative abundance varied depending on SAP (Hari et al., 2016b). Similarly, in acetate fed-MECs higher phylogenetic diversity was observed on the anode at positive SAP (0.37 V vs. SHE) than lower SAPs (-0.15, 0.09, and 0.02 V vs. SHE), which were mainly dominated by *G. sulfurreducens* (Torres et al., 2009). Taken together, these results suggest that the more energy available for growth at higher positive SAPs was likely captured by diverse microorganisms resulting in higher diversity.

In an earlier study, it was shown using a similar MEC setup and operated under the same conditions that multiple paths of carbon and electron flow (via acetate/H_2_ or acetate/formate) to electrical current could occur simultaneously during propionate oxidation in the anode of MECs regardless of the concentration tested (Hari et al., 2016a). In methanogenic systems, processing of substrates through multiple routes in parallel is essential for maintaining functional stability in response to organic overloading (Fernandez et al., 2000; Hashsham et al., 2000). In a similar fashion, this multiple paths of electron flow from the substrate in the anode of MECs should result in a higher functional stability of the system. Despite the high concentration (36 mM) of propionate used in the current study, the removal of propionate was still high (∼75%) in the PH reactors. Therefore, the anode of MECs could potentially be integrated with existing AD processes to improve propionate degradation and functional stability.

## Conclusion

Our findings indicated that MECs are functionally stable in performance regardless of the carbon source (acetate and propionate) and concentrations (0.5 g COD/L and 4 g COD/L) tested. *Geobacter* was the dominant genus at the anode of all the tested conditions. Their predominance was essential for maintaining relatively stable current density pattern despite frequent sampling of the anodic biofilms over time. The results of this study showed the potential of MECs as a viable alternative technology for anaerobic treatment of low and high strength synthetic solutions, containing acetate or propionate. However, defined solutions of acetate or propionate are not representative of the complexity of real wastewaters, and future studies focusing on the temporal dynamics of microbial communities and its correlation to system performance in MECs fed with real wastewater (containing various VFAs with differing concentrations) are needed to determine the robustness of MECs as an anaerobic treatment technology.

## Author Contributions

AH and PS conceptualized and designed the experiments. AH performed the experiments, analyzed data and wrote the manuscript. KV, KK, and PS helped in thoughtful discussion and revised the manuscript. SB helped in microbial community analysis.

## Conflict of Interest Statement

The authors declare that the research was conducted in the absence of any commercial or financial relationships that could be construed as a potential conflict of interest.

## References

[B1] Ambler J. R. Logan B. E. (2011). Evaluation of stainless steel cathodes and a bicarbonate buffer for hydrogen production in microbial electrolysis cells using a new method for measuring gas production. *Int. J. Hydrogen Energy* 36 160–166. 10.1016/j.ijhydene.2010.09.044

[B2] Aracic S. Semenec L. Franks A. E. (2014). Investigating microbial activities of electrode-associated microorganisms in real-time. *Front. Microbiol.* 5:663 10.3389/fmicb.2014.00663 PMC4246885 25506343

[B3] Boone D. R. Xun L. (1987). Effects of pH, temperature, and nutrients on propionate degradation by a methanogenic enrichment culture. *Appl. Environ. Microbiol.* 53 1589–1592. 16347387 10.1128/aem.53.7.1589-1592.1987 PMC203915

[B4] Cabezas A. Pommerenke B. Boon N. Friedrich M. W. (2015). *Geobacter, Anaeromyxobacter* and *Anaerolineae* populations are enriched on anodes of root exudate-driven microbial fuel cells in rice field soil. *Environ. Microbiol. Rep.* 7 489–497. 10.1111/1758-2229.12277 25683328

[B5] Cai W. Han T. Guo Z. Varrone C. Wang A. Liu W. (2016). Methane production enhancement by an independent cathode in integrated anaerobic reactor with microbial electrolysis. *Bioresour. Technol.* 208 13–18. 10.1016/j.biortech.2016.02.028 26913643

[B6] Call D. Logan B. E. (2008). Hydrogen production in a single chamber microbial electrolysis cell lacking a membrane. *Environ. Sci. Technol.* 42 3401–3406. 10.1021/es8001822 18522125

[B7] Caporaso J. G. Bittinger K. Bushman F. D. DeSantis T. Z. Andersen G. L. Knight R. (2010a). PyNAST: a flexible tool for aligning sequences to a template alignment. *Bioinformatics* 26 266–267. 10.1093/bioinformatics/btp636 19914921 PMC2804299

[B8] Caporaso J. G. Kuczynski J. Stombaugh J. Bittinger K. Bushman F. D. Costello E. K. (2010b). QIIME allows analysis of high-throughput community sequencing data. *Nat. Methods* 7 335–336. 10.1038/nmeth.f.303 20383131 PMC3156573

[B9] Chauhan A. Ogram A. Reddy K. (2004). Syntrophic-methanogenic associations along a nutrient gradient in the Florida Everglades. *Appl. Environ. Microbiol.* 70 3475–3484. 10.1128/AEM.70.6.3475-3484.2004 15184146 PMC427755

[B10] Dennis P. G. Guo K. Imelfort M. Jensen P. Tyson G. W. Rabaey K. (2013). Spatial uniformity of microbial diversity in a continuous bioelectrochemical system. *Bioresour. Technol.* 129 599–605. 10.1016/j.biortech.2012.11.098 23313735

[B11] Dumont M. G. Murrell J. C. (2005). Stable isotope probing—linking microbial identity to function. *Nat. Rev. Microbiol.* 3 499–504. 10.1038/nrmicro1162 15886694

[B12] Edgar R. C. (2010). Search and clustering orders of magnitude faster than BLAST. *Bioinformatics* 26 2460–2461. 10.1093/bioinformatics/btq461 20709691

[B13] Feng Y. Liu Y. Zhang Y. (2015a). Enhancement of sludge decomposition and hydrogen production from waste activated sludge in a microbial electrolysis cell with cheap electrodes. *Environ. Sci. Water Res. Technol.* 1 761–768. 10.1039/C5EW00112A

[B14] Feng Y. Zhang Y. Chen S. Quan X. (2015b). Enhanced production of methane from waste activated sludge by the combination of high-solid anaerobic digestion and microbial electrolysis cell with iron–graphite electrode. *Chem. Eng. J.* 259 787–794. 10.1016/j.cej.2014.08.048

[B15] Fernandez A. S. Hashsham S. A. Dollhopf S. L. Raskin L. Glagoleva O. Dazzo F. B. (2000). Flexible community structure correlates with stable community function in methanogenic bioreactor communities perturbed by glucose. *Appl. Environ. Microbiol.* 66 4058–4067. 10.1128/AEM.66.9.4058-4067.2000 10966429 PMC92259

[B16] Freguia S. Teh E. H. Boon N. Leung K. M. Keller J. Rabaey K. (2010). Microbial fuel cells operating on mixed fatty acids. *Bioresour. Technol.* 101 1233–1238. 10.1016/j.biortech.2009.09.054 19854639

[B17] Fu Q. Kobayashi H. Kawaguchi H. Vilcaez J. Wakayama T. Maeda H. (2013). Electrochemical and phylogenetic analyses of current-generating microorganisms in a thermophilic microbial fuel cell. *J. Biosci. Bioeng.* 115 268–271. 10.1016/j.jbiosc.2012.10.007 23164680

[B18] Gallert C. Winter J. (2008). Propionic acid accumulation and degradation during restart of a full-scale anaerobic biowaste digester. *Bioresour. Technol.* 99 170–178. 10.1016/j.biortech.2006.11.014 17197176

[B19] Goux X. Calusinska M. Lemaigre S. Marynowska M. Klocke M. Udelhoven T. (2015). Microbial community dynamics in replicate anaerobic digesters exposed sequentially to increasing organic loading rate, acidosis, and process recovery. *Biotechnol. Biofuels* 8 122 10.1186/s13068-015-0309-9 PMC4539856 26288654

[B20] Guo X. Liu J. Xiao B. (2013). Bioelectrochemical enhancement of hydrogen and methane production from the anaerobic digestion of sewage sludge in single-chamber membrane-free microbial electrolysis cells. *Int. J. Hydrogen Energy* 38 1342–1347. 10.1016/j.ijhydene.2012.11.087

[B21] Hari A. R. Katuri K. P. Gorron E. Logan B. E. Saikaly P. E. (2016a). Multiple paths of electron flow to current in microbial electrolysis cells fed with low and high concentrations of propionate. *Appl. Microbiol. Biotechnol.* 100 5999–6011. 10.1007/s00253-016-7402-2 26936773

[B22] Hari A. R. Katuri K. P. Logan B. E. Saikaly P. E. (2016b). Set anode potentials affect the electron fluxes and microbial community structure in propionate-fed microbial electrolysis cells. *Sci. Rep.* 6:38690 10.1038/srep38690 PMC5146674 27934925

[B23] Hashsham S. A. Fernandez A. S. Dollhopf S. L. Dazzo F. B. Hickey R. F. Tiedje J. M. (2000). Parallel processing of substrate correlates with greater functional stability in methanogenic bioreactor communities perturbed by glucose. *Appl. Environ. Microbiol.* 66 4050–4057. 10.1128/AEM.66.9.4050-4057.2000 10966428 PMC92258

[B24] Heidrich E. S. Edwards S. R. Dolfing J. Cotterill S. E. Curtis T. P. (2014). Performance of a pilot scale microbial electrolysis cell fed on domestic wastewater at ambient temperatures for a 12month period. *Bioresour. Technol.* 173 87–95. 10.1016/j.biortech.2014.09.083 25285764

[B25] Ishii S. Suzuki S. Norden-Krichmar T. M. Nealson K. H. Sekiguchi Y. Gorby Y. A. (2012). Functionally stable and phylogenetically diverse microbial enrichments from microbial fuel cells during wastewater treatment. *PLoS ONE* 7:e30495 10.1371/journal.pone.0030495 PMC3274515 22347379

[B26] Ishii S. I. Suzuki S. Norden-Krichmar T. M. Phan T. Wanger G. Nealson K. H. (2014). Microbial population and functional dynamics associated with surface potential and carbon metabolism. *ISME J.* 8 963–978. 10.1038/ismej.2013.217 24351938 PMC3996694

[B27] Ishii S. I. Suzuki S. Norden-Krichmar T. M. Tenney A. Chain P. S. Scholz M. B. (2013). A novel metatranscriptomic approach to identify gene expression dynamics during extracellular electron transfer. *Nat. Commun.* 4:1601 10.1038/ncomms2615 23511466

[B28] Ito T. Yoshiguchi K. Ariesyady H. D. Okabe S. (2011). Identification of a novel acetate-utilizing bacterium belonging to *Synergistes* group 4 in anaerobic digester sludge. *ISME J.* 5 1844–1856. 10.1038/ismej.2011.59 21562600 PMC3223300

[B29] Katuri K. Bettahalli N. M. S. Wang X. Matar G. Chisca S. Nunes P. S. (2016). A microfiltration polymer-based hollow fiber cathode as a promising advanced material for simultaneous recovery of energy and water. *Adv. Mater.* 28 9504–9511. 10.1002/adma.201603074 27615453

[B30] Katuri K. Werner C. M. Sandoval R. J. Chen W. Logan B. Lai Z. (2014). A novel anaerobic electrochemical membrane bioreactor (AnEMBR) with conductive hollow-fiber membrane for treatment of low-organic strength solutions. *Environ. Sci. Technol.* 48 12833–12841. 10.1021/es504392n 25310368

[B31] Kiely P. D. Cusick R. Call D. F. Selembo P. A. Regan J. M. Logan B. E. (2011). Anode microbial communities produced by changing from microbial fuel cell to microbial electrolysis cell operation using two different wastewaters. *Bioresour. Technol.* 102 388–394. 10.1016/j.biortech.2010.05.019 20554197

[B32] Klindworth A. Pruesse E. Schweer T. Peplies J. Quast C. Horn M. (2012). Evaluation of general 16S ribosomal RNA gene PCR primers for classical and next-generation sequencing-based diversity studies. *Nucleic Acids Res.* 41 e1 10.1093/nar/gks808 PMC3592464 22933715

[B33] Koch C. Harnisch F. (2016). Is there a specific ecological niche for electroactive microorganisms? *ChemElctroChem* 3 1282–1295. 10.1002/celc.201600079

[B34] Koch C. Popiel D. Harnisch F. (2014). Functional redundancy of microbial anodes fed by domestic wastewater. *ChemElectroChem* 1 1923–1931. 10.1002/celc.201402216

[B35] Kragelund C. Caterina L. Borger A. Thelen K. Eikelboom D. Tandoi V. (2007). Identity, abundance and ecophysiology of filamentous *Chloroflexi* species present in activated sludge treatment plants. *FEMS Microbiol. Ecol.* 59 671–682. 10.1111/j.1574-6941.2006.00251.x 17381520

[B36] Lee H.-S. Parameswaran P. Kato-Marcus A. Torres C. I. Rittmann B. E. (2008). Evaluation of energy-conversion efficiencies in microbial fuel cells (MFCs) utilizing fermentable and non-fermentable substrates. *Water Res.* 42 1501–1510. 10.1016/j.watres.2007.10.036 18035391

[B37] Lee H.-S. Torres C. I. Parameswaran P. Rittmann B. E. (2009). Fate of H2 in an upflow single-chamber microbial electrolysis cell using a metal-catalyst-free cathode. *Environ. Sci. Technol.* 43 7971–7976. 10.1021/es900204j 19921922

[B38] Lesnik K. Liu H. (2014). Establishing a core microbiome in acetate-fed microbial fuel cells. *Appl. Microbiol. Biotechnol.* 98 4187–4196. 10.1007/s00253-013-5502-9 24402416

[B39] Liu W. Cai W. Guo Z. Wang L. Yang C. Varrone C. (2016). Microbial electrolysis contribution to anaerobic digestion of waste activated sludge, leading to accelerated methane production. *Renew. Energy* 91 334–339. 10.1016/j.renene.2016.01.082

[B40] Logan B. E. Call D. Cheng S. Hamelers H. V. M. Sleutels T. H. J. A. Jeremiasse A. W. (2008). Microbial electrolysis cells for high yield hydrogen gas production from organic matter. *Environ. Sci. Technol.* 42 8630–8640. 10.1021/es801553z 19192774

[B41] Lu L. Xing D. Ren N. (2012a). Bioreactor performance and quantitative analysis of methanogenic and bacterial community dynamics in microbial electrolysis cells during large temperature fluctuations. *Environ. Sci. Technol.* 46 6874–6881. 10.1021/es300860a 22612779

[B42] Lu L. Xing D. Ren N. (2012b). Pyrosequencing reveals highly diverse microbial communities in microbial electrolysis cells involved in enhanced H2 production from waste activated sludge. *Water Res.* 46 2425–2434. 10.1016/j.watres.2012.02.005 22374298

[B43] Luo S. Guo W. Nealson K. H. Feng X. He Z. (2017). 13C pathway analysis for the role of formate in electricity generation by *Shewanella oneidensis* MR-1 using lactate in microbial fuel cells. *Sci. Rep.* 6:20941 10.1038/srep20941 PMC4751489 26868848

[B44] Ma J. Carballa M. Van De Caveye P. Verstraete W. (2009). Enhanced propionic acid degradation (EPAD) system: proof of principle and feasibility. *Water Res.* 43 3239–3248. 10.1016/j.watres.2009.04.046 19515396

[B45] Parameswaran P. Zhang H. Torres C. I. Rittmann B. E. Krajmalnik-Brown R. (2010). Microbial community structure in a biofilm anode fed with a fermentable substrate: the significance of hydrogen scavengers. *Biotechnol. Bioeng.* 105 69–78. 10.1002/bit.22508 19688868

[B46] Pullammanappallil P. C. Chynoweth D. P. Lyberatos G. Svoronos S. A. (2001). Stable performance of anaerobic digestion in the presence of a high concentration of propionic acid. *Bioresour. Technol.* 78 165–169. 10.1016/S0960-8524(00)00187-5 11333036

[B47] Ruiz V. Ilhan Z. E. Kang D.-W. Krajmalnik-Brown R. Buitrón G. (2014). The source of inoculum plays a defining role in the development of MEC microbial consortia fed with acetic and propionic acid mixtures. *J. Biotechnol.* 182 11–18. 10.1016/j.jbiotec.2014.04.016 24798298

[B48] Santoro C. Li B. Cristiani P. Squadrito G. (2013). Power generation of microbial fuel cells (MFCs) with low cathodic platinum loading. *Int. J. Hydrogen Energy* 38 692–700. 10.1016/j.ijhydene.2012.05.104

[B49] Shehab N. Li D. Amy G. L. Logan B. E. Saikaly P. E. (2013). Characterization of bacterial and archaeal communities in air-cathode microbial fuel cells, open circuit and sealed-off reactors. *Appl. Microbiol. Biotechnol.* 97 9885–9895. 10.1007/s00253-013-5025-4 23775270

[B50] Siegert M. Li X.-F. Yates M. D. Logan B. E. (2014). The presence of hydrogenotrophic methanogens in the inoculum improves methane gas production in microbial electrolysis cells. *Front. Microbiol.* 5:778 10.3389/fmicb.2014.00778 PMC4295556 25642216

[B51] Stams A. J. Plugge C. M. (2009). Electron transfer in syntrophic communities of anaerobic bacteria and archaea. *Nat. Rev. Microbiol.* 7 568–577. 10.1038/nrmicro2166 19609258

[B52] Tandishabo K. Iga Y. Tamaki H. Nakamura K. Takamizawa K. (2012). Characterization of a novel *Coprothermobacter* sp. strain IT3 isolated from an anaerobic digester – hydrogen production and peptidase profiles at higher temperature. *J. Environ. Conserv. Eng.* 41 753–761. 10.5956/jriet.41.753

[B53] Torres C. I. Krajmalnik-Brown R. Parameswaran P. Marcus A. K. Wanger G. Gorby Y. A. (2009). Selecting anode-respiring bacteria based on anode potential: phylogenetic, electrochemical, and microscopic characterization. *Environ. Sci. Technol.* 43 9519–9524. 10.1021/es902165y 20000550

[B54] Vanwonterghem I. Jensen P. D. Dennis P. G. Hugenholtz P. Rabaey K. Tyson G. W. (2014). Deterministic processes guide long-term synchronised population dynamics in replicate anaerobic digesters. *ISME J.* 8 2015–2028. 10.1038/ismej.2014.50 24739627 PMC4184015

[B55] Vargas I. T. Albert I. U. Regan J. M. (2013). Spatial distribution of bacterial communities on volumetric and planar anodes in single-chamber air-cathode microbial fuel cells. *Biotechnol. Bioeng.* 110 3059–3062. 10.1002/bit.24949 23616357

[B56] Wang Q. Garrity G. M. Tiedje J. M. Cole J. R. (2007). Naive Bayesian classifier for rapid assignment of rRNA sequences into the new bacterial taxonomy. *Appl. Environ. Microbiol.* 73 5261–5267. 10.1128/AEM.00062-07 17586664 PMC1950982

[B57] Werner C. M. Katuri K. Logan B. E. Amy G. L. Saikaly P. E. (2016). Graphene-coated hollow fiber membrane as the cathode in anaerobic electrochemical membrane bioreactors – Effect of configuration and applied voltage on performance and membrane fouling. *Environ. Sci. Technol.* 50 4439–4447. 10.1021/acs.est.5b02833 26691927

[B58] Yamada T. Imachi H. Ohashi A. Harada H. Hanada S. Kamagata Y. (2007). *Bellilinea caldifistulae* gen. nov., sp. nov. and *Longilinea arvoryzae* gen. nov., sp. nov., strictly anaerobic, filamentous bacteria of the phylum *Chloroflexi* isolated from methanogenic propionate-degrading consortia. *Int. J. Syst. Evol. Microbiol.* 57 2299–2306. 10.1099/ijs.0.65098-0 17911301

[B59] Zhu X. Yates M. D. Hatzell M. C. Ananda Rao H. Saikaly P. E. Logan B. E. (2014). Microbial community composition is unaffected by anode potential. *Environ. Sci. Technol.* 48 1352–1358. 10.1021/es404690q 24364567

