# Supplementary material for: Temporal Microbial Community Dynamics in Microbial Electrolysis Cells – Influence of Acetate and Propionate Concentration
Source: Front Microbiol. 2017 Jul 20;8:1371. doi: 10.3389/fmicb.2017.01371 (PMC5517442; doi:10.3389/fmicb.2017.01371)
Supplement: Supplementary file 1 [file Data_Sheet_1.PDF]

frontiers

# Supporting Information

# Temporal microbial community dynamics in microbial electrolysis cells – influence of acetate and propionate concentration

**Ananda Rao Hari [a]*, Krishnaveni Venkidusamy [b], Krishna Katuri[a], Samik Bagchi[c], and Pascal E. Saikaly[a]***

[a]King Abdullah University of Science and Technology, Biological and Environmental Sciences and Engineering Division, Water Desalination and Reuse Research Center, Thuwal 23955-6900, Saudi Arabia

[b]Centre for Environmental Risk Assessment and Remediation (CERAR), University of South Australia, Mawson Lakes, South Australia, Australia

[c]University of Kansas, Department of Civil, Environmental, and Architectural Engineering, Lawrence, KS, USA 66045

*** Correspondence:**
Corresponding Authors Dr. Ananda Rao Hari (hari.anandarao@kaust.edu.sa) and Prof. Pascal Saikaly (pascal.saikaly@kaust.edu.sa)

frontiers

# Supporting Information

**Table S1.** The relative abundance of bacterial phylotypes classified to the lowest level possible (class, order, family or genus) for A-reactors: A, anode. Bacterial phyla representing less than 1% of the relative abundance presented as others in the table. The numbers after the symbols represent the sampling periods.

| Taxa level (class) | Taxa level (order, family or genus ) | A(AL-0) | A(AL-10) | A(AL-30) | A(AL-50) | A(AL-70) | A(AH-0) | A(AH-15) | A(AH-35) | A(AH-70) |
|---|---|---|---|---|---|---|---|---|---|---|
| *Deltaproteobacteria* | *Geobacter* | 0.5±0.3 | 50.37±4.2 | 66.52±3.7 | 55.46±3.5 | 35.56±4 | 0.8±0.4 | 52.73±2.1 | 47.27±3.6 | 45.7±4.3 |
| | *Desulfovibrio* | 0.2±0.1 | 2.59±1.6 | 1.38±0.43 | 3.46±2.5 | 9.35±3.8 | 0.5±0.3 | 1.70±1 | 3.58±2.1 | 4.77±2.7 |
| | *Desulfuromonadales* | 4.51±1.9 | 0.5±0.3 | 0.00 | 0.00 | 0.00 | 1.09±0.7 | 0.5±0.4 | 0.3±0.2 | 0.00 |
| | *Pelobacteraceae* | 0.00 | 1.18±0.7 | 1.81±0.5 | 4.19±2.3 | 4.62±2.2 | 23.28±7.4 | 4.14±1.9 | 1.17±0.6 | 0.7±0.5 |
| *Clostridia* | *Coprothermobacter* | 57.83±8.7 | 20.71±5.6 | 11.39±4.9 | 8.53±4.3 | 8.56±4.8 | 33.98±5.5 | 9.84±3.7 | 14.31±5.4 | 11.07±3.3 |
| | *Sedimentibacter* | 0.00 | 0.00 | 0.00 | 0.00 | 0.00 | 0.6±0.4 | 1.74±0.8 | 2.03±1 | 0.7±0.5 |
| | *Soehngenia* | 1.68±0.9 | 2.81±2.2 | 0.5±0.3 | 2±1.1 | 1±0.6 | 5.53±2.5 | 3.58±2.7 | 4.3±2.3 | 2.58±2.5 |
| | *Clostridiaceae* | 0.00 | 0.00 | 0.5±0.4 | 0.7±0.4 | 0.7±0.6 | 0.00 | 0.3±0.2 | 0.4±o.3 | 0.3±0.1 |
| *Synergistia* | *Dethiosulfovibrionaceae* | 4.48±2.6 | 5.70±2.9 | 3.15±2.1 | 3.90±2 | 3.60±1.8 | 4.34±2.2 | 1.70±1 | 1.32±0.7 | 0.7±0.4 |
| | *Synergistaceae* | 4.33±2.3 | 1.02±0.5 | 1.56±0.8 | 0.7±0.6 | 1.52±0.7 | 1.90±1.1 | 0.7±0.5 | 0.5±0.3 | 0.8±0.5 |
| *Betaproteobacteria* | *Rhodocyclaceae* | 0.00 | 0.00 | 0.00 | 0.3±0.2 | 0.2±0.1 | 1.36±0.7 | 0.7±0.5 | 1.82±0.9 | 0.5±0.3 |
| | *Thauera* | 0.00 | 0.00 | 0.2±0.2 | 0.4±0.3 | 0.2±0.1 | 0.00 | 0.3±0.3 | 0.6±0.4 | 0.5±0.5 |
| *Gammaproteobacteria* | *Pseudomonadaceae* | 0.3±0.1 | 0.5+0.4 | 0.3±0.1 | 3±1.7 | 3.3±2.1 | 2.13±1.2 | 1.47±0.8 | 1.6±1 | 0.5±0.3 |
| | *Pseudomonas* | 0.2±0.2 | 0.30 | 0.70 | 2.10 | 8.60 | 0.30 | 0.20 | 1.30 | 4.00 |
| | *Shewanella* | 0.00 | 0.00 | 0.00 | 0.5±0.5 | 0.8±0.5 | 0.00 | 1.47±0.8 | 1.59±0.7 | 0.3±0.2 |
| *Anaerolineae* | *Anaerolinaceae* | 4.04±1.9 | 2.29±1.7 | 0.5±0.4 | 0.3±0.1 | 0.00 | 0.00 | 0.5±0.4 | 1.35±0.7 | 9.90±4.3 |
| *Bacteroidia* | *Bacteroidales* | 0.3±0.2 | 0.2±0.1 | 0.00 | 0.00 | 0.00 | 2.06±1.5 | 0.7±0.5 | 1.91±1 | 0.5±0.3 |
| | *Porphyromonadaceae* | 0.00 | 0.00 | 0.00 | 0.00 | 0.00 | 0.00 | 0.6±0.4 | 1±0.6 | 3±1.8 |
| Others | | 21.6±5.1 | 11.29±3.4 | 11.46±4.1 | 14.44±3.8 | 21.95±3.6 | 22.09±4.3 | 17.08±2.6 | 13.57±4.5 | 13.43±3.3 |

# Supporting Information

**Table S2.** The relative abundance of bacterial phylotypes classified to the lowest level possible (class, order, family or genus) for A-reactors: S, suspension. Bacterial phyla representing less than 1% of the relative abundance presented as others in the table. The numbers after the symbols represent the sampling periods.

| Taxa level (class) | Taxa level (order, family or genus ) | S(AL-0) | S(AL-10) | S(AL-30) | S(AL-50) | S(AL-70) | S(AH-0) | S(AH-15) | S(AH-35) | S(AH-70) |
|---|---|---|---|---|---|---|---|---|---|---|
| *Deltaproteobacteria* | *Geobacter* | 0.00 | 18.59±3.7 | 28.72±2.2 | 29.72±2.9 | 14.12±3.3 | 0.00 | 30.37±1.9 | 9.97±5.7 | 45.31±3.1 |
| | *Desulfovibrio* | 0.00 | 1.21±0.7 | 3.09±1.2 | 14.41±2.8 | 6.91±3.1 | 0.00 | 2.17±0.9 | 4.87±2.2 | 5.53±3.2 |
| | *Pelobacteraceae* | 0.00 | 0.3±0.1 | 1.60±0.5 | 2.23±1 | 0.6±0.4 | 1.41±0.6 | 1.47±0.8 | 0.3±0.3 | 0.2±0.1 |
| *Clostridia* | *Coprothermobacter* | 47.11±6.3 | 31.38±4.9 | 3.17±1.4 | 3.40±1 | 0.4±0.2 | 39.15±4.6 | 4.17±1.7 | 11.67±2.9 | 1.08±0.5 |
| | *Sedimentibacter* | 0.3±0.2 | 0.7±0.4 | 4.90±1.8 | 1.83±0.7 | 0.6±0.4 | 0.4±0.4 | 7.32±3.3 | 5.09±2.3 | 0.7±0.4 |
| | *Soehngenia* | 2.95±1.8 | 2.13±1.1 | 0.4±0.3 | 0.2±0.2 | 0.00 | 12.31±3.6 | 1.52±0.8 | 0.4±0.3 | 0.3±0.3 |
| | *Desulfosporomusa* | 0.4±0.3 | 0.7±0.5 | 5.81±2.6 | 2.63±1.9 | 8.07±3.1 | 0.2±0.1 | 1.04±0.5 | 6.29±2.9 | 1.76±0.8 |
| | *Clostridiaceae* | 1.17±0.5 | 0.7±0.4 | 0.4±0.4 | 0.2±0.1 | 0.3±0.3 | 0.00 | 0.8±0.6 | 2.81±1.5 | 0.5±0.4 |
| | *Oscillospira* | 0.00 | 0.00 | 0.00 | 0.2±0.2 | 0.3±0.1 | 0.00 | 0.5±0.3 | 0.3±0.3 | 0.00 |
| *Synergistia* | *Dethiosulfovibrionaceae* | 5.16±3 | 2.86±2.1 | 0.6±0.5 | 8.53±2.5 | 1.99±1.1 | 7.37±3.4 | 0.5±0.5 | 1.84±0.7 | 0.7±0.4 |
| | *Synergistaceae* | 6.92±3.6 | 1.99±0.9 | 0.5±0.3 | 1.13±0.7 | 0.7±0.5 | 5.76±2.2 | 5.8±3.5 | 0.8±0.6 | 7±3.6 |
| *Betaproteobacteria* | *Betaproteobacteria* | 1±0.6 | 8±3.9 | 7.20±3.7 | 3.13±1.3 | 1.35±0.7 | 0.3±0.2 | 1.00±0.6 | 3.68±1.4 | 0.5±0.3 |
| | *Dechloromonas* | 0.00 | 0.00 | 0.00 | 0.00 | 0.00 | 0.00 | 0.6±0.5 | 0.7±0.6 | 6.37±3.8 |
| | *Rhodocyclaceae* | 0.00 | 0.00 | 0.00 | 0.00 | 0.00 | 0.5±0.4 | 2.61±1.1 | 18.66±5.3 | 2.08±0.9 |
| | *Alcaligenaceae* | 0.00 | 0.00 | 0.00 | 0.00 | 0.00 | 0.00 | 0.00 | 0.4±0.2 | 0.3±0.2 |
| *Gammaproteobacteria* | *Pseudomonadaceae* | 0.7±0.7 | 5.2±2.7 | 16.5±4.3 | 1.1±0.6 | 31.3±4.2 | 4.83±2.2 | 16.1±4.1 | 0.9±0.6 | 14±4.7 |
| | *Pseudomonas* | 0.4±0.4 | 4.2±2.4 | 3.1±1.6 | 2±1.1 | 7.8±3.3 | 0.3±0.2 | 3.3±1.5 | 0.5±0.4 | 0.3±0.3 |
| | *Shewanella* | 0.00 | 0.3±0.3 | 6.38±3.3 | 0.4±0.3 | 0.4±0.4 | 0.00 | 4.97±2.6 | 0.8±0.6 | 4.00±2.2 |
| *Anaerolineae* | *Anaerolinaceae* | 1.97±0.9 | 1.23±0.7 | 0.2±0.1 | 0.00 | 0.00 | 1.37±0.8 | 0.4±0.3 | 2.42±1.4 | 2.07±1 |
| *Bacteroidia* | *Bacteroidales* | 0.00 | 0.00 | 0.00 | 0.00 | 0.00 | 0.00 | 0.00 | 0.4±0.3 | 0.3±0.2 |
| | *Porphyromonadaceae* | 0.00 | 0.00 | 0.00 | 0.00 | 0.00 | 0.00 | 0.7±0.6 | 2.6±1.5 | 0.5±0.5 |
| | *Parabacteroides* | 0.00 | 0.00 | 1.41±0.8 | 0.5±0.4 | 0.3±0.3 | 0.00 | 0.6±0.5 | 4±2.1 | 0.4±0.3 |
| | *Dysgonomonas* | 0.00 | 0.00 | 0.00 | 0.00 | 0.00 | 0.00 | 0.00 | 0.3±0.2 | 0.6±0.6 |
| *Actinobacteria* | *Leucobacter* | 3.82±2.2 | 3.06±1.7 | 0.5±0.5 | 0.00 | 0.00 | 0.00 | 0.00 | 0.00 | 0.00 |
| Others | | 30±5.7 | 20.88±4.5 | 17.09±4 | 30.91±6.1 | 27.72±5.3 | 27.77±6.4 | 18.21±5.8 | 26.04±5.5 | 16.01±4.7 |

# Supporting Information

**Table S3.** The relative abundance of bacterial phylotypes classified to the lowest level possible (class, order, family or genus) for P-reactors: A, anode. Bacterial phyla representing less than 1% of the relative abundance presented as others in the table. The numbers after the symbols represent the sampling periods.

| Taxa level (class) | Taxa level (order, family or genus ) | A (PL-0) | A (PL-10) | A (PL-30) | A (PL-50) | A (PL-70) | A (PH-0) | A (PH-20) | A (PH-35) | A (PH-70) |
|---|---|---|---|---|---|---|---|---|---|---|
| *Deltaproteobacteria* | *Geobacter* | 1.38±0.5 | 72.71±5.1 | 70.73±7.8 | 66.14±5.5 | 38.78±2.3 | 11.52±1.8 | 48.74±6.1 | 23.25±2.7 | 41.86±6.2 |
| | *Desulfovibrio* | 0.6±0.4 | 1.83±0.8 | 3.43±1.5 | 11.39±3.2 | 9.22±3.8 | 0.0 | 1.58±1 | 0.4±0.2 | 1.20±0.4 |
| | *Desulfuromonadales* | 2.58±0.7 | 0.0 | 0.0 | 0.0 | 0.0 | 0.0 | 0.0 | 0.0 | 0.0 |
| | *Pelobacteraceae* | 10.76±3.2 | 0.0 | 0.0 | 0.0 | 0.0 | 2.32±0.8 | 0.0 | 0.0 | 0.0 |
| | *Syntrophobacter* | 0.0 | 0.0 | 0.0 | 0.0 | 0.0 | 0.0 | 0.0 | 0.3±0.2 | 0.3±0.1 |
| *Clostridia* | *Coprothermobacter* | 24.87±7.4 | 2.79±1.2 | 2.79±1.2 | 1.98±0.7 | 2.30±1.1 | 19.83±4.2 | 12.78±2.9 | 5.27±2.3 | 1.41±1 |
| | *Sedimentibacter* | 2.46±1.1 | 0.0 | 1.28±0.5 | 0.4±0.3 | 1.15±0.6 | 0.5±0.3 | 5.67±2.4 | 2.54±1.1 | 1.61±0.9 |
| | *Syntrophomonas* | 0.0 | 0.0 | 0.0 | 0.0 | 0.0 | 0.0 | 0.5±0.4 | 1.99±0.9 | 0.5±0.3 |
| | *Soehngenia* | 3.74±1.7 | 0.96±0.3 | 1.32±0.5 | 0.0 | 0.0 | 1.46±0.6 | 1.33±0.4 | 0.4±0.1 | 0.3±0.1 |
| | *Clostridiales* | 0.4±0.4 | 0.0 | 0.0 | 0.0 | 0.0 | 0.3±0.3 | 0.6±0.2 | 3.46±1.5 | 5±1.8 |
| *Synergistia* | *Dethiosulfovibrionaceae* | 6.61±2.8 | 6.10±2.7 | 5.04±2.1 | 5.77±1.5 | 9.64±2.7 | 7.72±2.5 | 3.31±0.8 | 22.82±5.3 | 2.86±1.5 |
| | *Synergistaceae* | 4.93±2.1 | 0.67±0.2 | 0.0 | 0.2±0.3 | 5.2±1.8 | 7.19±1.8 | 0.5±0.2 | 4.1±1.7 | 1.53±0.8 |
| *Betaproteobacteria* | *Dechloromonas* | 1.3±0.9 | 0.0 | 2.19±0.9 | 0.5±0.2 | 0.4±0.2 | 2.97±1.5 | 2.25±1.2 | 0.7±0.5 | 0.6±0.2 |
| | *Rhodocyclaceae* | 0.0 | 0.0 | 0.0 | 0.0 | 0.0 | 0.0 | 0.0 | 0.0 | 0.0 |
| | *Thauera* | 0.0 | 0.0 | 0.0 | 0.0 | 0.0 | 0.0 | 0.0 | 0.3±0.1 | 4.52±2.4 |
| *Gammaproteobacteria* | *Pseudomonadaceae* | 0.93±0.3 | 0.2±0.1 | 0.3±0.3 | 0.3±0.2 | 1.25±0.7 | 1.56±0.4 | 0.4±0.2 | 0.4±0.2 | 3.8±1.1 |
| | *Shewanella* | 0.0 | 0.0 | 0.0 | 0.0 | 0.0 | 0.7±0.3 | 5.42±1.8 | 0.5±0.2 | 5.73±2.3 |
| *Anaerolineae* | *Anaerolinaceae* | 15.44±3.9 | 5.19±2.1 | 5.15±2.3 | 5.17±2.3 | 14.98±3.2 | 15.55±3.6 | 6.02±2.4 | 13.69±3.7 | 4.98±1.9 |
| *Bacteroidia* | *Porphyromonadaceae* | 2.38±1.1 | 0.0 | 0.0 | 1.76±0.7 | 1.25±0.8 | 1.58±0.9 | 0.4±0.3 | 1.17±0.8 | 1.49±0.5 |
| | *Bacteroidales* | 0.3±0.3 | 0.0 | 0.0 | 0.0 | 0.0 | 1.30±0.7 | 0.3±0.1 | 0.2±0.1 | 0.4±0.2 |
| *Actinobacteria* | *Leucobacter* | 2.62±1.2 | 0.0 | 0.0 | 0.0 | 0.0 | 2.13±0.9 | 0.6±0.3 | 1.07±0.6 | 1.2±0.8 |
| Others | | 18.7±3.5 | 9.5±2.8 | 7.7±2.8 | 6.4±2.3 | 15.8±2.5 | 23.3±5.2 | 9.6±3.7 | 17.4±4.1 | 20.7±4.5 |

# Supporting Information

**Table S4**. The relative abundance of bacterial phylotypes classified to the lowest level possible (class, order, family or genus) for P-reactors: S, suspension. Bacterial phyla representing less than 1% of the relative abundance presented as others in the table. The numbers after the symbols represent the sampling periods.

| Taxa level (class) | Taxa level (order, family or genus ) | S (PL-0) | S (PL-10) | S (PL-30) | S (PL-50) | S (PL-70) | S (PH-0) | S (PH-20) | S (PH-35) | S (PH-70) |
|---|---|---|---|---|---|---|---|---|---|---|
| *Deltaproteobacteria* | *Geobacter* | 0.0 | 44.19±4.7 | 10.13±3.3 | 9.59±2.6 | 2.62±1 | 3.28±1.5 | 2.61±1.4 | 6.63±2.5 | 12.88±3.5 |
| | *Desulfovibrio* | 0.0 | 6.44±2.9 | 3.31±1.9 | 6.77±2.9 | 8.43±2.7 | 1±0.4 | 3.30±1.3 | 0.9±0.2 | 0.4±0.2 |
| | *Desulfuromonadales* | 0.0 | 1.38±0.9 | 0.5±0.3 | 0.0 | 0.0 | 0.3±0.1 | 0.3±0.1 | 0.3±0.1 | 2.77±1.4 |
| | *Pelobacteraceae* | 0.0 | 0.0 | 2.45±0.9 | 0.0 | 0.0 | 0.6±0.4 | 0.4±0.2 | 0.3±0.1 | 0.2±0.1 |
| *Clostridia* | *Coprothermobacter* | 19.28±2.7 | 0.5±0.3 | 1.33±1 | 0.4±0.2 | 0.3±0.1 | 17.70±4.4 | 4.54±2.1 | 1.63±0.7 | 0.4±0.2 |
| | *Sedimentibacter* | 0.7±0.2 | 10.26±3.3 | 2.86±1.2 | 3.58±1.6 | 1.96±0.7 | 0.5±0.4 | 14.14±4.3 | 7.65±2.6 | 7.53±2.2 |
| | *Soehngenia* | 8.98±2.1 | 0.0 | 0.0 | 0.0 | 0.0 | 6.44±2.6 | 1±0.4 | 0.4±0.1 | 0.3±0.1 |
| | *Clostridiales* | 0.9±0.6 | 7.87±2.6 | 0.7±0.3 | 3.6±0.9 | 0.6±0.2 | 0.5±0.2 | 2.49±1.6 | 3.6±1.1 | 4.6±1.9 |
| | *Desulfosporomusa* | 0.0 | 0.0 | 0.0 | 3.36±1.7 | 3.84±1.5 | 0.2±0.1 | 0.0 | 0.9±0.4 | 0.0 |
| | *Syntrophomonas* | 0.0 | 0.0 | 0.0 | 0.0 | 0.0 | 0.4±0.2 | 0.0 | 1.83±0.8 | 0.0 |
| *Synergistia* | *Dethiosulfovibrionaceae* | 11.30±2.8 | 2.93±1.4 | 1.84±0.7 | 11.56±3.1 | 11.90±2.8 | 16.7±5.1 | 2.80±1.2 | 29.68±3.9 | 5.52±2.3 |
| | *Synergistaceae* | 8.79±3.2 | 0.6±0.5 | 1.01±0.6 | 0.3±0.1 | 12.08±3.7 | 13.9±3.7 | 1.80±0.8 | 13.62±3.1 | 8.91±3.9 |
| *Betaproteobacteria* | *Betaproteobacteria* | 2.52±1.4 | 0.5±0.3 | 3.33±1.3 | 1.32±0.5 | 0.7±0.5 | 0.3±0.1 | 1.93±0.7 | 0.3±0.1 | 0.0 |
| | *Rhodocyclaceae* | 0.5±0.3 | 0.0 | 1.04±0.4 | 0.7±0.5 | 0.5±0.3 | 0.7±0.3 | 1.74±0.5 | 0.0 | 0.0 |
| | *Thauera* | 0.0 | 0.0 | 0.0 | 0.5±0.2 | 0.6±0.4 | 0.5±0.2 | 0.3±0.1 | 0.0 | 0.0 |
| | *Dechloromonas* | 0.8±0.5 | 6.06±3.7 | 41.32±6.3 | 18.51±4.5 | 2.15±1.7 | 1.49±1.1 | 37.45±5.2 | 3.36±1.2 | 5.80±2.7 |
| *Gammaproteobacteria* | *Pseudomonadaceae* | 0.6±0.4 | 2.96±1.1 | 12.19±3.2 | 4.70±2.2 | 1.21±0.4 | 12.5±3.5 | 5.7±2.5 | 0.5±0.2 | 0.4±0.2 |
| | *Shewanella* | 0.5±0.2 | 0.0 | 9.45±3.9 | 0.4±0.3 | 28.95±5.7 | 0.6±0.3 | 0.5±0.2 | 0.9±0.8 | 0.5±0.4 |
| *Anaerolineae* | *Anaerolineae* | 8.73±2.7 | 0.6±0.4 | 0.7±0.3 | 1.01±0.8 | 10.49±3.5 | 7.04±2.1 | 0.7±0.4 | 2.04±1.4 | 5.81±2.2 |
| *Bacteroidia* | *Porphyromonadaceae* | 2.39±1.4 | 1.49±0.6 | 1.1±0.4 | 7.11±2.8 | 2.06±1.6 | 3.08±1.5 | 3.5±1.8 | 1.52±0.4 | 19.23±4.1 |
| | *Bacteroidales* | 2.24±1.6 | 4.87±1.8 | 1.6±0.9 | 3.93±2.5 | 0.7±0.5 | 3.49±2.8 | 0.6±0.5 | 2.44±0.9 | 0.0 |
| | *Dysgonomonas* | 0.7±0.5 | 4.39±2.2 | 0.8±0.7 | 0.6±0.5 | 0.0 | 1.4±0.8 | 0.5±0.2 | 0.3±0.3 | 0.0 |
| *Alphaproteobacteria* | *Telmatospirillum* | 0.0 | 0.0 | 0.3±0.1 | 0.2±0.1 | 0.0 | 0.0 | 0.0 | 0.3±0.1 | 4.80±2.4 |
| Others | | 31±4.2 | 5±2.4 | 4±1.4 | 22±4.8 | 11±3.5 | 7±2.6 | 14±3.1 | 21±3.9 | 20±2.7 |

# Supporting Information

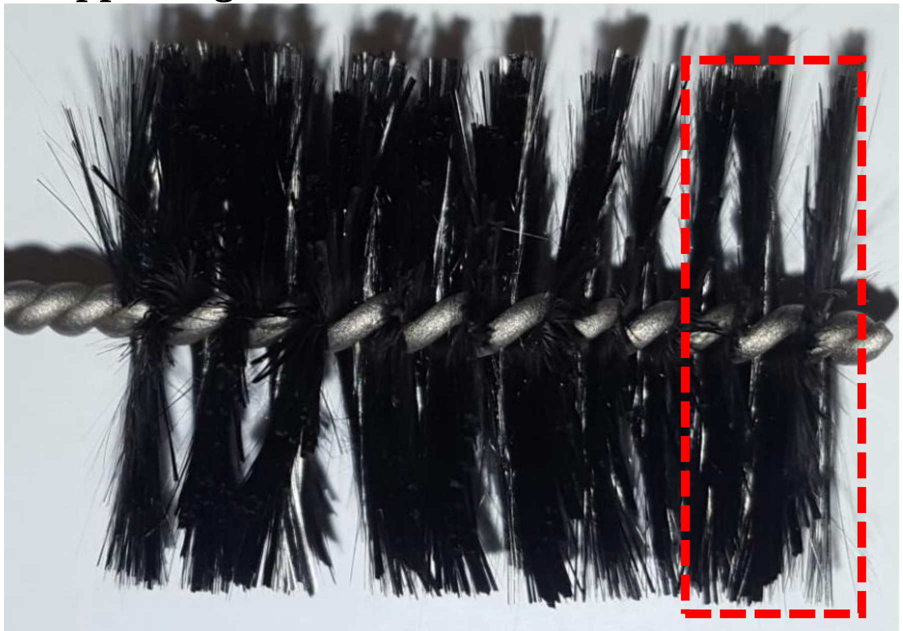

**Supplementary Figure S1.** Photo of the brush anode used in this study. The dashed rectangle represents the section of the anode that was sampled for microbial analysis.

# Supporting Information

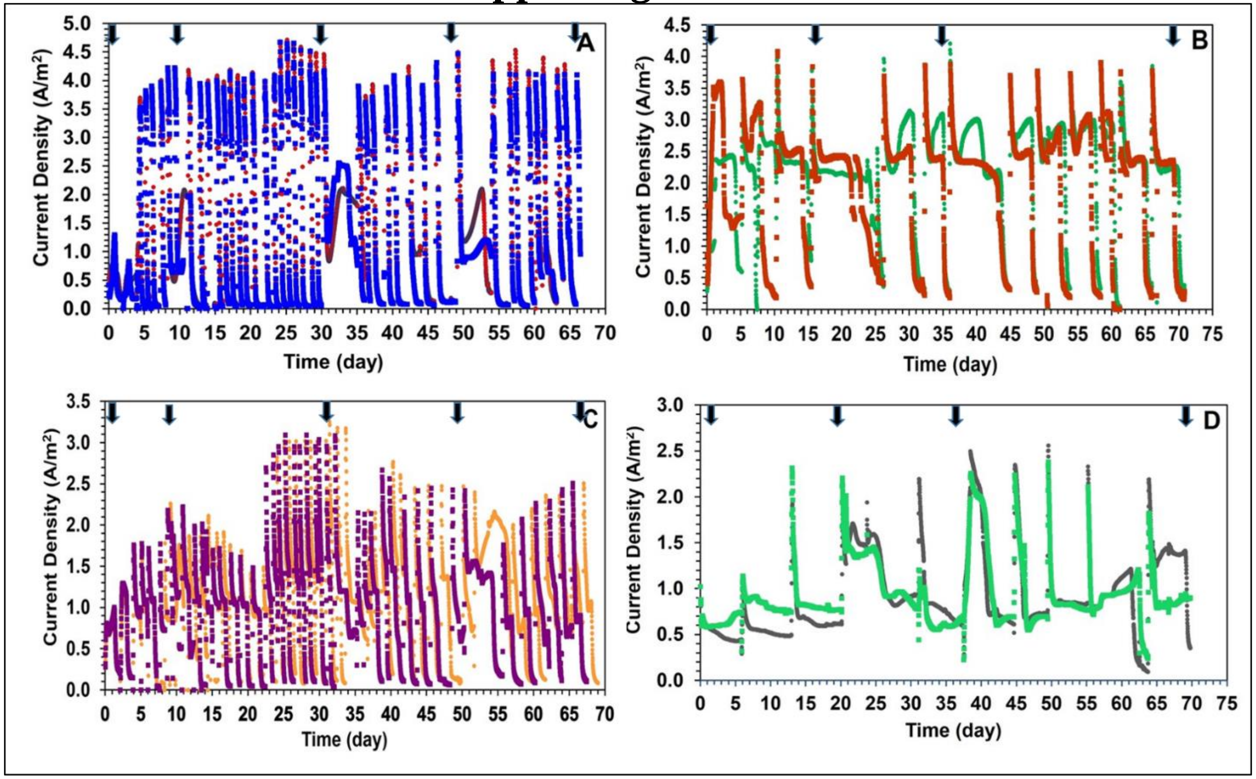


**Supplementary Figure S2.** Current density profile in duplicate MECs fed with low and high concentrations of acetate and propionate. (A) MECs fed with low acetate concentration (AL); (B) MECs fed with high acetate concentration (AH); (C) MECs fed with low propionate concentration (PL); (D) MECs fed with high propionate concentration (PH). Arrows indicate anode biofilm sampling for DNA extraction.

# Supporting Information

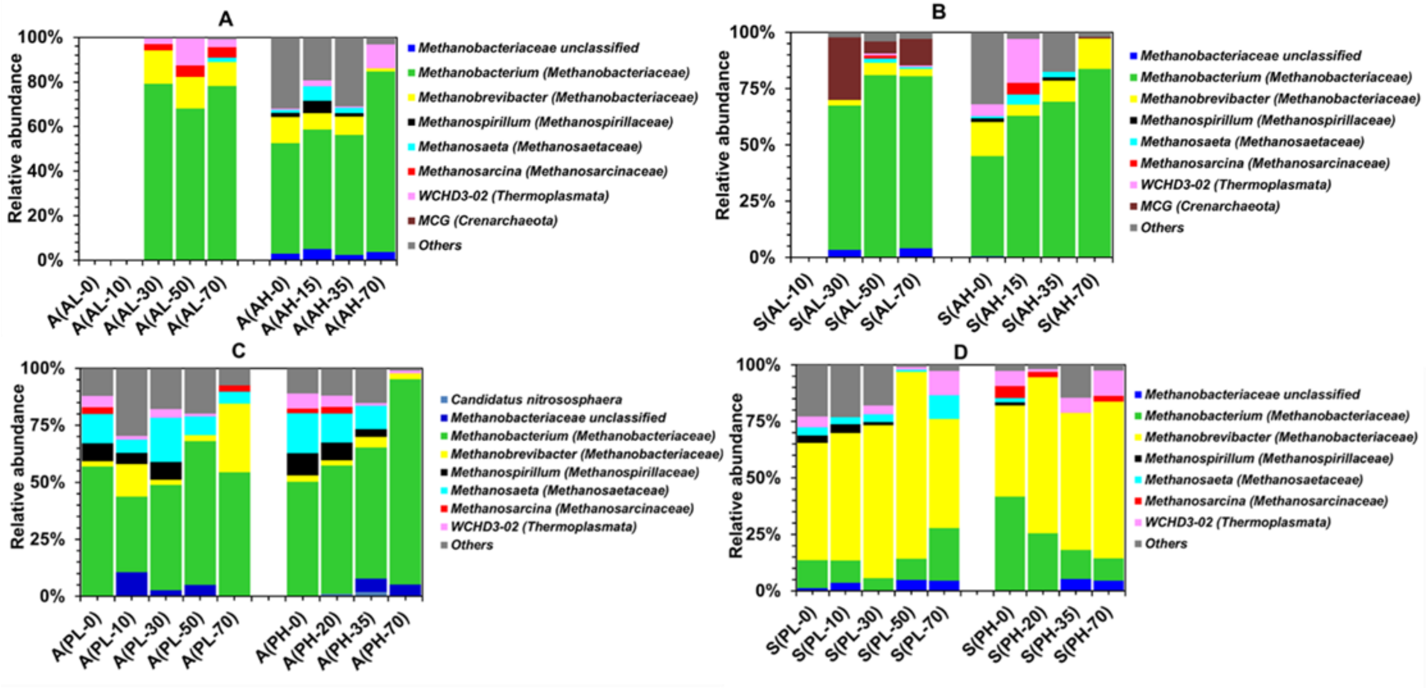


**Supplementary Figure S3.** Relative abundance of archaeal phylotypes to the lowest classification level possible (class, family and genus) for the anode of A-reactors (A), suspension of A-reactors (B), anode of P-reactors (C) and suspension of P-reactors (D). A, anode and S, suspension. Archaeal phylotypes representing less than 1% of the relative abundance are classified as others. The taxa level shown on the right-hand side of the panel represents class, family and genus. Samples A (AL-0 and 10) and S (AL-0 and 10) failed to amplify.
